# Evolution of dependoparvoviruses across geological timescales—implications for design of AAV-based gene therapy vectors

**DOI:** 10.1093/ve/veaa043

**Published:** 2020-05-22

**Authors:** Evin Hildebrandt, Judit J Penzes, Robert J Gifford, Mavis Agbandje-Mckenna, Robert M Kotin

**Affiliations:** University of Massachusetts Medical School, Department of Microbiology and Physiological Systems, Gene Therapy Center, 55 Lake Ave. North, Worcester, MA 01655, USA; University of Florida, Department of Biochemistry and Molecular Biology, Center for Structural Biology, The McKnight Brain Institute, 1200 Newell Drive, Gainesville, Florida, 32610, USA; MRC-University of Glasgow Centre for Virus Research, Genomics & Bioinformatics, Sir Michael Stoker Building Garscube Campus, 464 Bearsden Road, Glasgow G61 1QH, Scotland, UK; University of Florida, Department of Biochemistry and Molecular Biology, Center for Structural Biology, The McKnight Brain Institute, 1200 Newell Drive, Gainesville, Florida, 32610, USA; University of Massachusetts Medical School, Department of Microbiology and Physiological Systems, Gene Therapy Center, 55 Lake Ave. North, Worcester, MA 01655, USA

**Keywords:** paleovirology, gene therapy, adeno-associated virus (AAV), endogenous viral element (EVE), dependoparvovirus

## Abstract

Endogenous viral elements (EVEs) are genetic remnants of viruses that have integrated into host genomes millions of years ago and retained as heritable elements passed on to offspring until present-day. As a result, EVEs provide an opportunity to analyse the genomes of extinct viruses utilizing these genomic viral fossils to study evolution of viruses over large timescales. Analysis of sequences from near full-length EVEs of dependoparvoviral origin identified within three mammalian taxa, Whippomorpha (whales and hippos), Vespertilionidae (smooth-nosed bats), and Lagomorpha (rabbits, hares, and pikas), indicates that distinct ancestral dependoparvovirus species integrated into these host genomes approximately 77 to 23 million years ago. These ancestral viruses are unique relative to modern adeno-associated viruses (AAVs), and distinct from extant species of genus *Dependoparvovirus*. These EVE sequences show characteristics previously unseen in modern, mammalian AAVs, but instead appear more similar to the more primitive, autonomously replicating and pathogenic waterfowl dependoparvoviruses. Phylogeny reconstruction suggests that the whippomorph EVE orthologue derives from exogenous ancestors of autonomous and highly pathogenic dependoparvovirus lineages, believed to have uniquely co-evolved with waterfowl birds to present date. In contrast, ancestors of the two other mammalian orthologues (Lagomorpha and Vespertilionidae) likely shared the same lineage as all other known mammalian exogenous AAVs. Comparative *in silico* analysis of the EVE genomes revealed remarkable overall conservation of AAV *rep* and *cap* genes, despite millions of years of integration within the host germline. Modelling these proteins identified unexpected variety, even between orthologues, in previously defined capsid viral protein (VP) variable regions, especially in those related to the three- and fivefold symmetry axes of the capsid. Moreover, the normally well-conserved phospholipase A2 domain of the predicted minor VP1 also exhibited a high degree of sequence variance. These findings may indicate unique biological properties for these virus ‘fossils’ relative to extant dependoparvoviruses and suggest key regions to explore within capsid sequences that may confer novel properties for engineered gene therapy vectors based on paleovirology data.

## 1. Introduction 

Parvoviruses are small, non-enveloped viruses, with an icosahedral capsid and a linear, single-stranded DNA genome. *Parvoviridae* is classified into two subfamilies; *Densovirinae* and *Parvovirinae*, based on distinct host ranges infecting either invertebrates or vertebrates, respectively (Cotmore et al. 2019). The *Parvovirinae* subfamily includes members of the *Dependoparvovirus* genus, of which the vast majority require co-infection with a helper DNA virus to render the cell permissive for replication. Host-species infected by dependoparvoviruses represent a remarkably broad range encompassing all major amniote vertebrate lineages from squamate reptiles to primates (Pénzes and Benkő 2014; Pénzes et al. 2015; Lau et al. 2017; Cotmore et al. 2019). Among the dependoparvoviruses, adeno-associated viruses (AAVs) are considered non-pathogenic, whereas others, for example, the autonomous waterfowl viruses, can cause severe pathology, such as Derzsy’s disease (Zádori et al. 1995). For helper-associated, as well as autonomous dependoparvoviruses, determinants of pathogenicity are poorly understood (Kailasan, Agbandje-McKenna, and Parrish 2015). Most members of the *Dependoparvovirus* genus have overcome their S-phase linked replication limitations by using large DNA viruses, such as adenoviruses or herpesvirus, as helper viruses during concurrent infection. In particular, human adeno-associated virus 2 (AAV2) was originally identified as a contaminant in adenovirus stocks, as indicated by their name (Atchison, Casto, and Hammon 1965).

The dependoparvovirus genome contains two major open reading frames (ORFs), which encode four non-structural Rep proteins and three structural capsid viral proteins (VPs), via alternative splicing and alternative translation initiation. The mRNA populations of AAVs are transcribed in temporal order from three promoters, located at map units 5, 19, and 40, in the AAV2 genome and utilize a common polyadenylation site, directly upstream of the right ITR (Mouw and Pintel 2000; Qiu, Cheng, and Pintel 2006). A third alternative ORF within the *cap* gene encodes the assembly activating protein, AAP, required for efficient capsid assembly (Sonntag et al. 2011).

Capsid structures of AAV serotypes are known to be highly conserved, possessing icosahedral symmetry assembled from 60 monomers composed of the minor VP1 and VP2, and major VP3, in a 1:1:10 ratio, respectively. The VPs are overlapping with VP1 containing a unique N-terminal sequence (VP1u) that encodes a phospholipase A2 (PLA2) enzyme required for infection. The ordered structure of the overlapping VP3 region contains conserved β-strands, forming a β-barrel ‘jelly roll’ core linked by surface-exposed variable loops that confer the virion surface morphology (Chapman and Agbandje-Mckenna 2006). The surface loops encompass nine variable regions (VR-I to VR-IX) that determine AAV serotype-specific functions (Chapman and Agbandje-Mckenna 2006). Characteristically, the AAV capsid possesses a channel with a pore-like opening at each fivefold symmetry axis, protrusions surrounding the threefold symmetry axes, and a depression at the twofold axes (Cotmore and Tattersall 2005; Cotmore, Hafenstein, and Tattersall 2010). A raised capsid region between the two- and fivefold axes is referred to as the two/fivefold wall.

AAVs have proven utility as gene therapy vectors that are safe and efficacious for delivery and expression of therapeutic genes due to non-pathogenicity and ability to infect both dividing and non-dividing cells. However, utility of AAV-based vectors can be compromised when patients have previously been exposed to wild-type AAV serotypes that are closely related to therapeutic capsids used in gene therapy. As a result, these individuals are ineligible for therapy utilizing conventional AAV vectors. Estimates indicate that nearly 70 per cent of the population have pre-existing antibodies to common AAV strains and this prevalence of pre-existing immunity remains an obstacle for widespread therapeutic use (Mingozzi and High 2013). Therefore, characterization of novel AAV genotypes would facilitate the development of AAV vectors that are less likely to be neutralized due to patient’s prior exposure to circulating AAVs and could allow the identification of novel capsid proteins that possess unique biological properties, such as tissue tropism or biodistribution. Such capsids are highly desired to increase the utility and functional range for targeted tissue delivery of gene therapy vectors. To help address these needs, we utilized a paleovirology-based approach employing endogenous viral element (EVE) sequences as an innovative resource for identifying new AAV-genotypes and capsid motifs not found in known extant viruses.

EVEs are DNA sequences derived from viruses that once circulated in a host population, but integrated into cells of the host germline, thus they to become endogenous sequences within host genomes (Katzourakis and Gifford 2010; Smith et al. 2016). Due to the absence of viral specimens in physical fossil records, EVEs offer an alternative method to study viral evolution across geologic time by functioning as surrogates for traditional paleontological remains. These genomic fossils can be inherited and maintained for millions of years. Thus, EVEs provide a snapshot of the ancestral viral sequence at the time of integration and preserve sequence variation from viral lineages that may not be represented by modern viruses circulating in populations today.

EVEs can be derived from a variety of diverse viral families with RNA genomes, like retroviruses, or DNA genomes such as the *Parvoviridae*, which is composed of various genera including *Ambidensovirus*, *Protoparvovirus*, *Amdoparvovirus*, and *Dependoparvovirus* (Belyi, Levine, and Skalka 2010; Katzourakis and Gifford 2010; Arriagada and Gifford 2014; Smith et al. 2016; Pénzes et al. 2018). *In silico* mining of eukaryotic whole-genome reference sequences has identified EVEs derived from endogenous parvoviral elements (EPVs) in a broad range of eukaryotic species (Zhu et al. 2018). Family Parvoviridae has been well characterized for its ability to integrate within genomes of the host (Belyi, Levine, and Skalka 2010; Liu et al. 2011; Arriagada and Gifford 2014; François et al. 2016; Pénzes et al. 2018, 2019; Callaway et al. 2019). This phenomenon, however, is unbiased throughout the family. The vast majority of EPVs present in invertebrate genomes originated from exogenous members of genus *Ambidensovirus* of subfamily Densovirinae (Liu et al. 2011; François et al. 2016). Interestingly, a divergent vertebrate-infecting parvoviral lineage, currently dubbed as ‘Chapparvovirus’ has been shown to harbour closely related EPVs in the genomes of several arthropods but absent in vertebrate genomes (Pénzes et al. 2019). As for subfamily *Parvovirinae*, members of only three genera have been shown be capable of fixation in the host germline, namely *Amdoparvovirus*, *Protoparvovirus*, and *Dependoparvovirus* (Arriagada and Gifford 2014; Smith et al. 2016; Pénzes et al. 2019). Numerous EPV derived from dependoparvoviruses have been identified, however most EVEs are fragments spanning only portions of viral genomes. Nevertheless, previous studies have identified several EPV that encompass complete or nearly complete virus genomes (Katzourakis and Gifford 2010; Smith et al. 2016). In this study, we investigated three genome-length EPV sequences derived from ancient dependoparvoviruses that share high degrees of sequence similarity to modern AAV species. Tissue samples and cell lines from multiple species belonging to three mammalian orders, namely the Cetacea clade of Cetartiodactyla (whales and dolphins), Lagomorpha (rabbits, hares, and pikas), and Chiroptera (bats) were acquired to establish the prevalence of the EPV within these species, estimate the age of their integrated virus, and compare viral sequence diversity across these orders. By examining these ancient viral sequences via a combination of *in vitro* and *in silico* approaches we draw inferences into the biology and evolution of dependoparvoviruses by comparison to modern viruses.

## 2. Results

### 2.1 Prevalence and age of cetacean EPV (EPV-Dp.1-Whippomorpha)

Presence of the cetacean EPV (Dp.1-Whippo) first identified in the *Tursiops truncatus* reference genome (NIST Tur_tru v1, Assembly GCF_001922835.1) was confirmed in all cetacean specimens analysed (Supplementary Table S1) and the *Hippopotamus amphibius* reference genome (ASM299558v1). The Dp.1-Whippo element is integrated into an intron of the Pax5 gene of the Whippomorpha suborder (*Cetacea* and hippopotamuses) (Waddell, Okada, and Hasegawa 1999) of the Cetartiodactyla, but is absent from the equivalent Pax5 region of artiodactyls based on comparative sequence analysis to the *Bos taurus* (Bos_taurus_UMD_3.1.1, Assembly GCA_000003055.5) reference genome. Integration of this EPV into the ancestral whippomorph germline therefore must have occurred after the ruminantia and whippomorpha lineages split, but before divergence of cetaceans and hippos. Based on time-calibrated phylogenies for speciation of cetacea and hippos suggests a window of 60 to 55 million years ago (MYA), in which the ancestral dependoparvovirus represented by the Dp.1 sequence would have circulated and infected progenitors of the Whippomorpha suborder.

Comparing alignments of Dp.1 sequences from various whippomorph species shows *rep* and *cap* genes have undergone varying degrees of disruption among the species analysed. The most intact copy is found in the minke whale (*Balaenoptera bonaerensis*) and the most degraded within the oceanic dolphins (family Delphinidae). The *rep* sequences in hippo and minke whale EVEs contained only four frameshifts. Terminal deletion of the *rep* gene was observed in all species, with 267 nt being deleted from the 5′-end, and 312 nt of the 3′-end (large Rep-coding sequence) relative to the most closely related dependoparvovirus, namely goose parvovirus, with which the minke whale element Rep displays 57 per cent amino acid (aa) identity. The least intact *rep* was found in the Dp.1 sequence of the killer whale (*Orcinus orca*), with six frameshifts and five stop codons. A broader number of post-integration mutations were observed in the whippomorph *cap* gene. Despite these indels, the entire aa sequence of VP1 could be reconstructed, with the exception of the hippo EPV where the C-terminus past VR-IX is missing (Supplementary Fig. S1). The *cap* ORF was also disrupted by four to six frameshifts and one to six stop codons, depending on species. Prediction of promoters identified a region homologous to p40 in the whippomorph EPVs, however, the previously mentioned N-term deletion within *rep* simultaneously deleted the putative region containing a p5 promoter homologue.

**Figure 1.**
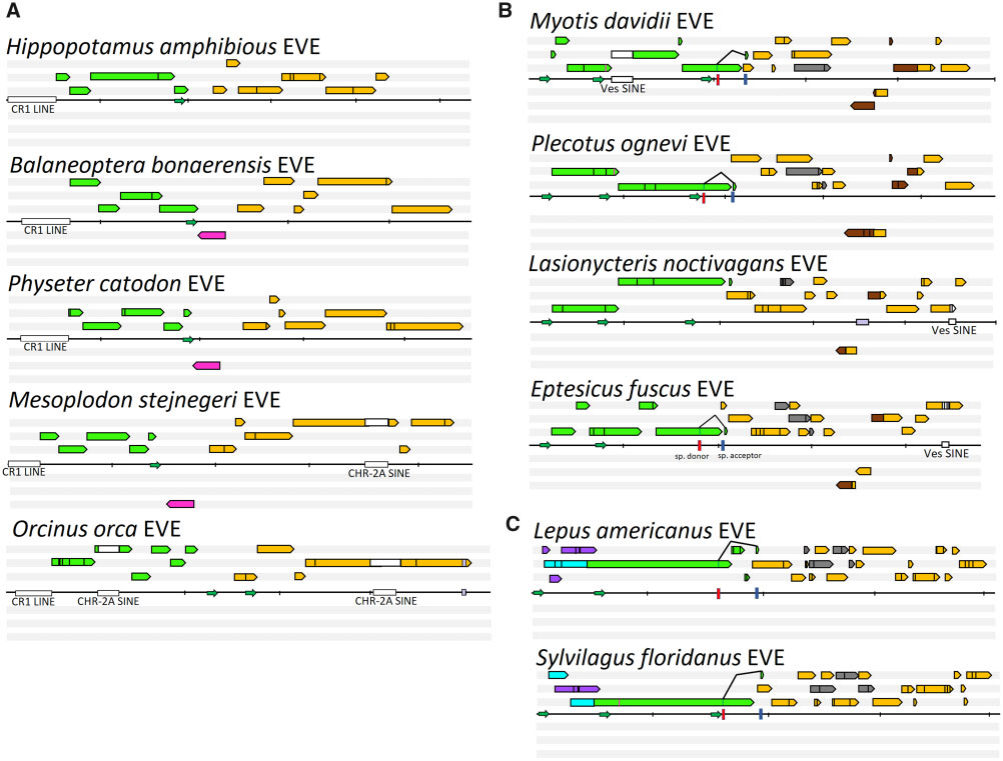
The annotation of dependoparvoviral EVE representatives, shown in six frames, from the three orders investigated. EVEs of Whippomorpha are presented by panel A, Chiroptera by panel B, and Lagomorpha by panel C. Reading frames are indicated by colored arrows, where green signifies homologues of rep, orange of cap and grey of AAP. Repeated stretches are highlighted in brown. Frames encoding proteins of host origin are shown in magenta. Homologous of the two, distinct, large Rep N-term encoding ORFs of lagomorph entries are marked in purple (frame A) and cyan (frame B). Retrotransposons are represented by white, insertions of unknown origin by lilac squares. The Pax5 homologue of whippomorphs is indicated in magenta. Promoters, which were identified with scores higher than 0.95 of 1.00 are marked by small green arrows. Predicted splice sites are shown as colored bars, red for donor, blue for acceptor ones. Each vertical dash represents 1,000 nucleotides. Stop codons are represented by the vertical black bars inside the ORF-symbolizing arrows. The predicted translational start sites of Rep52/40 are marked by the magenta bars.

Through comparative analysis of Dp.1-Whippo sequences, multiple insertions of additional exogenous DNA within EPVs were identified (Fig. 1). One insertion, exclusive to cetaceans, involved a rearrangement incorporating 120 aa from the Pax-5 gene in opposite orientation to the EPV coding sequences. Another small insertion of unknown origin (∼150 nt) integrated into the 3′ end of *cap* from *Delphinidae* species. Additional integrations derived from retrotransposal origins were also identified (Fig. 1 and Supplementary Fig. S1). A long-interspersed element integration of the chicken repeat class, could be identified in the whippomorpha EPV, resulting in deletion of the 5′ region of *rep*. Furthermore, multiple short-interspersed elements (SINEs) of the CHRS-2 family were identified within cetacean EPVs. Members of the *Delphinidae* clade had two copies of the CHRS-2 SINE, present in both the *rep* and *cap* genes (Fig. 1). Conversely, beaked whales (*Mesoplodon stejnegeri* and *Ziphius cavirostris*) possessed a single CHRS-2 SINE integrated into the *cap* region of their EPV while baleen whales, represented by the minke whale (*B.bonaerensis*) and basal toothed whales, represented by the sperm whale (*Physeter catodon*), and hippos had no copies of the SINE within their respective EPV sequences (Fig. 1). Timescale estimates for speciation of baleen, toothed, and beaked whales indicate that the *cap*-localized SINE first integrated into the common ancestor of beaked whales and dolphins between approximately 34.7 and 31.2 MYA (McGowen, Spaulding, and Gatesy 2009), followed by integration of a second CHRS-2 SINE into the Rep EPV region of *Delphinidae* species approximately 31.2 to 24.7 MYA.

Two cetacean cell lines (derived from *Balaenoptera physalus* and *T.truncatus*) (generously provided by San Diego Global) were chosen as representatives for cetacean species lacking any SINE integrations in their EPV (*B.physalus*) and cetaceans with SINE integrations in both *rep* and *cap* regions (*T.truncatus).* Following transcriptome sequencing of cell lines, only two sequencing reads were mapped to the EPV locus of *T.truncatus* but both reads corresponded to the CHR-2 SINE sequence nested within the EPV’s VP region and not the EVE sequence itself. CHR-2 sequences are known to occur at numerous loci within cetacean genomes and share incredibly high levels of sequence identity, often differing only by a few nucleotides. These two mapped reads are not suggestive for expression of the EVEs, but likely result from sequencing errors leading to altered mapping of other CHR-2 SINE elements from elsewhere in the genome to the EVE loci’s SINE (Nikaido et al. 2001). In contrast, RNA sequencing (RNA-seq) analysis of *B.physalus* results in 13 sequencing reads that mapped across both *rep* and *cap* portions of their EPV (Supplementary Fig. S2A), indicating that the relatively intact cetacean EVEs (i.e. *B.physalus*) appear to retain a level of EVE expression, unlike the dolphin EVE containing SINE integrations within their EPV.

### 2.2 Prevalence and age of *EPV-Dp.2-Vespertilionidae*

A genome-length EPV has previously been identified *in silico* from the genome of *Myotis* bats (Katzourakis and Gifford 2010). A combination of screening ten species by PCR of extracted DNA and *in silico* analysis of reference genomes available through National Center for Biotechnology Information (NCBI) for fourteen species, revealed eight chiroptera species, all members of *Vespertilionidae*, carry orthologous copies of this EPV, which is fixed within an intron of the KIF6 gene (Supplementary Table S2). Comparison of the reference genome of *Miniopterus natalensis*, a member of the Miniopteridae bats and the sister clade to Vespertilionidae, indicates that Miniopteridae bats lack integration of an EPV within the KIF6 locus. This limits the estimated time of integration for the KIF6-EVE to the most recent common ancestor (MRCA) of vesper bats based on radiation of these families to no greater than 49 to 38 MYA: the divergence dates for Miniopteridae and Vespertilionidae, but earlier than 30 to 19 MYA, during the speciation of the *Eptesicus* and *Myotis* genera of bats (Miller-Butterworth et al. 2007).

Protein-encoding ORFs inferred for *Dp.2-Vespertilionidae* reveal that the coding sequence of the *rep* gene was relatively well preserved compared to *cap*, a trend also seen in the Dp.1-Whippo EPV (Fig. 1, Supplementary Figs S1 and S3). Within *rep*, the number of observed frameshifts varied from between two (genera *Lasinycteris* and *Plecotus*) to six (genus *Myotis*) and the in-frame stop codons from one to as many as five when comparing *Myotis* versus *Plecotus*, respectively (Fig. 1B). Despite the various disruptions of the *rep* ORF, both *rep* promoters could be identified with high reliability (0.95–1.00 out of 1.00). The presence of an additional, small ORF encoding a 10-aa-long C-terminal extension, analogous to the AAV Rep68/40 C-terminal tails, could be identified as well as both splice sites with high confidence (>0.95 on the donor and 0.78–0.98 on the acceptor site), thus suggesting that the ancestral virus harboured a similar transcription pattern to that of modern primate AAVs (Fig. 1B). Despite the ubiquitous presence of the 3′-*cap* ORF, splice sites were not detected within the *Lasionycteris noctivagans* EVE (Fig. 1B). An EPV promoter homologous to p40 of AAV2, was absent from both *Eptesicus* species EVEs, despite preservation in the other six vesper species. Although the Vespertilionidae EVEs were predicted to be slightly younger than the whippomorph EVEs, the preservation of the *cap* gene was significantly poorer, including as many as eleven frameshifts and up to four stop codons, depending on the species (Fig. 1B). A unique phenomenon within this family was the presence of frequent, repeated nt stretches inserted into *cap*, of which the longest one, encoding VR-III to VR-VI, was found in a reverse direction (Fig. 1B). Repeated elements inferred from the original *cap* and the reverse-inserted fragment had accumulated various point mutations, altering as many as seven aa in genus *Myotis*. This reverse insertion was entirely absent from the *Eptesicus andinus* EVE. Unlike the whippomorph EVEs, the entire coding sequence of the AAP could be identified, with the exception of the *L.noctivagans* EVE, where only a fragment, corresponding to the conserved core encoding region was identified.

Similar to the *Dp.*1 element in cetaceans, *Dp.2* vesper bat element sequences contain multiple retrotransposon insertions (originating from the Ves SINE family) (Fig. 1B). A heavily truncated version of this SINE was revealed in the *cap* 5′ encoding region of the EVEs of genera *Eptesicus* and *Lasionycteris*, whereas out of *Myotis* only *M.davidii* included the full-length, probably recently integrated Ves SINE. Mapping of RNA reads sequenced from cell lines of the EVE-positive species identified transcripts originating from the EVE sequences. The bat cell line derived from *Myotis velifer incautus* had only two reads mapped with 100 per cent identity match to the *Myotis* reference EVE sequence within the approximately 4 kb EVE region. These two overlapping reads mapped to a 239 bp region of the Vesper EVE’s *cap* C-term region of the VPs.

### 2.3 Prevalence and age of lagomorph EPV (EPV-Dp.3-Lagomorpha)

EPVs reflecting full-length, viral genome insertions were present in all species of the Leporidae screened (Fig. 1C, Supplementary Table S3, Figs S4 and S5). Further investigation determined that this element, which is located within an intergenic region between the NUPL2 and GPNMB genes on chromosome 10 of the domestic rabbit genome (OryCun2.0), was also present in a more distantly related lagomorph species, the American pika (*Ochotona princeps*), although in this species only a portion of the *rep* gene remains. These results indicate that the EPV integration occurred in the germline of the MRCA of all lagomorphs. Based on evolutionary timescales of lagomorphs, the MRCA would have been infected with a circulating AAV prior to divergence of Ochotonidae and Leporidae families ∼50 MYA (Ge et al. 2013). Comparison to the mouse genome (*Mus musculus*) show that this element is exclusive to the order Lagomorpha, indicating that this EPV derives from an ancient dependoparvovirus that was incorporated into the germline of the common ancestor of extant lagomorphs after the Rodentia/Lagomorpha split, estimated to have occurred between 66 and 47 MYA (Ge et al. 2013; Benton et al. 2015).

**Figure 2.**
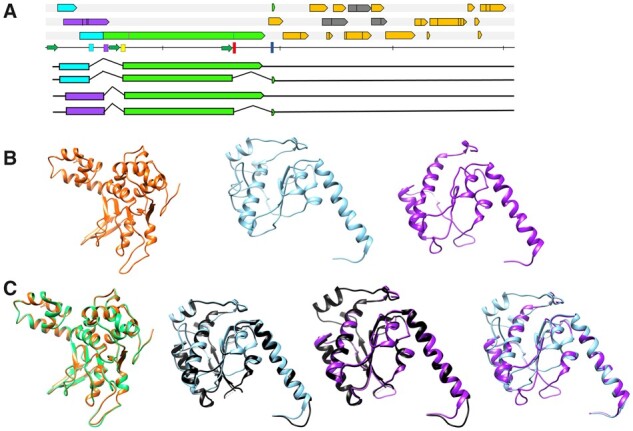
The hypothesized expression strategy and homology modelling of the Leporidae endogenous dependoparvovirus-like element Rep represented by the element of *S.floridanus*. Panel A shows the hypothetic transcription pattern from the upstream most promoter, homologous to p5 of AAV2. Both ancestral reading frames with the potential of encoding the N-terminal region of the large Rep78/68, namely frame A (purple) and frame B (cyan) could be possibly spliced to the *rep* gene (green), using donor sites marked by the purple and cyan bars, respectively. Homologues of the *cap* gene are shown in orange, homologues of the AAP frame in grey, and the vertical bars represent stop codons. The blue and red bars indicate the donor- and acceptor sites used to express the C-terminal truncated versions of Rep, that is Rep68/40. Hypothesized transcripts are mapped below the genome map. Panel B displays homology models of the Rep DNA-binding regions encoded by both ORF A and B as well as of Rep40 of the *S.floridanus* element. From left to right the Rep40, the ‘cyan’ N-terminus and the purple N-terminus Rep78/68 homology models are shown. Panel C shows these models superimposed with the respective crystal structure model of AAV2 or with each other. From left to right: *S.floridanus* Rep40 (orange) with AAV2 Rep40 (green); *S.floridanus* Rep78/68 N-term ‘cyan’ (blue) with that of AAV2 (black); *S.floridanus* Rep78/68 N-term ‘purple’ (purple) with that of AAV2 (black); *S.floridanus* Rep78/68 N-term ‘purple’ (purple) and ‘cyan’ superimposed.

In addition to preserving the full-length *rep* and *cap* genes, Leporidae EPVs retained identifiable dependoparvoviral *cis*-regulatory elements, that is three dependoparvovirus promoters (two for *rep* and one for *cap*), and splice donor and acceptor sites of the Rep68/Rep40 homologues (Fig. 1C). All eight Dp.3-Lago sequences analysed were predicted to have the capacity to encode an intact and potentially functional Rep40 homologue, while the most well-preserved *Sylvilagus floridanus* EPV was expected to encode Rep40 and Rep52 homologues (Fig. 1C). Furthermore, there appear to be remnants of two possible ORFs that encode Rep78 and Rep68 (Fig. 2A). Although both frames have been disrupted to various degrees, there are two ubiquitous splice donor sites, as well as an acceptor site, capable of restoring reading frame A or B in the *rep* transcript. The hypothesized transcription mechanism of the p5 homologue from the most intact *S.floridanus* element is presented by (Fig. 2). As previously seen with whippomorph and chiroptera EPVs, the lagomorph’s EPV *cap* ORFs are noticeable more degraded than *rep* ORFs, displaying fifteen to sixteen frameshifts and nine to ten stop codons. The AAP reading-frame could be reconstructed with deletions in its hydrophobic region, as well as the T/S rich region (Naumer et al. 2012).

RNA-seq analysis revealed numerous transcripts from the domestic rabbit (*Oryctolagus cuniculus*) cell line RK13 (ATCC^®^ CCL-37) mapped to both *rep* and *cap* regions of the rabbit EPV. Thirty-one sequencing reads mapped to the *rep* region and were distributed across the entire length of *rep*,whereas four sequencing reads mapped to the 5′ end of *cap*(Supplementary Fig. S2B). This illustrates expression of Dp.3-Lago sequences within the transcriptome of rabbits, with a bias particularly towards greater number of transcripts derived from the more intact and conserved rep portion of the EPV.

### 2.4 Structural diversity and long-term evolution of dependoparvovirus capsids

An important aim of our study was to explore the diversity of ancient dependoparvovirus genotypes to gain insight into the structural composition of ancestral virus capids. Although none of the EPVs analysed within this study encoded fully intact ORFs, utilizing a large set of orthologous EPVs from numerous lagomorph, cetacean or vespertilionid species provided the means to infer ancestral dependoparovovirus ORFs based on sequence comparisons of species-specific EPVs from these three mammalian taxa (Fig. 1). Once ancestral ORFs were reconstructed, representative EVEs for the three taxonomic orders were subjected to homology modelling using extant dependoparvoviruses as templates.

Despite numerous indels and mutations disrupting all of these endogenous ORFs to varying degrees, the derived aa sequences appeared to harbour similar percentage of aa identity as their exogenous counterparts within genus Dependoparvovirus (Table 1). All three orthologues shared the smallest percentage of identical residues with the Rep and Cap of squamate reptilian dependoparvoviruses, which is also true in case of the exogenous mammalian and avian members of the genus. Of these three elements, the Leporidae EVE consistently displayed the lowest identity scores when compared to exogenous dependoparvoviral Rep and Cap protein sequences.

**Table 1.** Percent identity for derived protein sequences between the *rep* (blue) and the *cap* (yellow) gene for species of genus *Dependoparvovirus* and the three dependoparvovirus-like EVEs.

	AAV2	AAV5	Bat AAV	California sealion AAV	Avian AAV	Goose PV	Snake AAV	Bearded dragon PV	Leporidae EVE	Whippomorpha EVE	Vespertilionidae EVE
AAV2		59%	63%	56%	59%	56%	54%	55%	43%	48%	53%
AAV5	66%		57%	53%	56%	55%	51%	53%	44%	47%	48%
Bat AAV	54%	50%		57%	57%	56%	57%	57%	40%	48%	50%
California sealion AAV	61%	64%	53%		55%	52%	50%	52%	38%	44%	45%
Avian AAV	56%	52%	49%	51%		58%	53%	56%	40%	46%	46%
Goose PV	49%	51%	44%	49%	49%		54%	54%	37%	47%	45%
Snake AAV	39%	38%	38%	37%	39%	35%		70%	35%	44%	43%
Bearded dragon PV	37%	37%	35%	35%	37%	34%	58%		38%	45%	44%
Leporidae EVE	43%	41%	40%	40%	39%	33%	27%	26%		34%	32%
Whippomorpha EVE	50%	51%	45%	51%	51%	57%	38%	38%	33%		37%
Vespertilionidae EVE	52%	50%	42%	47%	43%	39%	32%	30%	35%	37%	

Inferred Rep protein sequences derived from Leporidae EPVs could be reliably aligned to homologous regions of extant dependoparvoviral Rep proteins for their two large Rep protein N-terminus variants (A and B) (Supplementary Fig. S4A and B). Using these sequences, structural homology models were constructed based on the AAV2 Rep DNA-binding motif (N-terminal region) as a template (Fig. 2). A model could also be generated for the leporid Rep40 helicase region (Fig. 2). Overall, the derived aa sequence of the reconstructed *cap* suggested all these elements originated from ancestors with characteristically parvovirus-like capsids due to the presence of the PLA2 domain and surface-exposed VRs. These regions, however, frequently included indels compared to AAV2, the prototypical virus of the *Dependoparvovirus* genus (Supplementary Table S4). In the VP1u region, a glycine-rich stretch could be detected in all Cetacea elements, upstream the of PLA2 domain (Supplementary Fig. S1). The PLA2 domain, normally conserved among genera of exogenous parvoviruses, showed an unexpected variety of point mutations and indels (Supplementary Figs S1, S3, S4 and Table S4) In the case of Whippomorpha, PLA2 orthologues contain insertions of 31 aa that disrupt the catalytic core of the inferred proteins encoded by Dp.1-*Lipotes* and the Dp.1-*Hippotamus* elements (Supplementary Fig. S1 and Table S4). Within Vespertilionidae, a four-aa-long deletion affected the C-terminal conserved region of the catalytic core only in the Dp.2 sequence from *E.andinus*, the entity with an overall more disrupted *cap* coding sequence (Supplementary Fig. S3 and Table S4). In the examples of Dp.3-Lagomorpha the canonical HD motif of the catalytic core was uniformly absent due to a six or seven aa deletion in the region.

**Figure 3.**
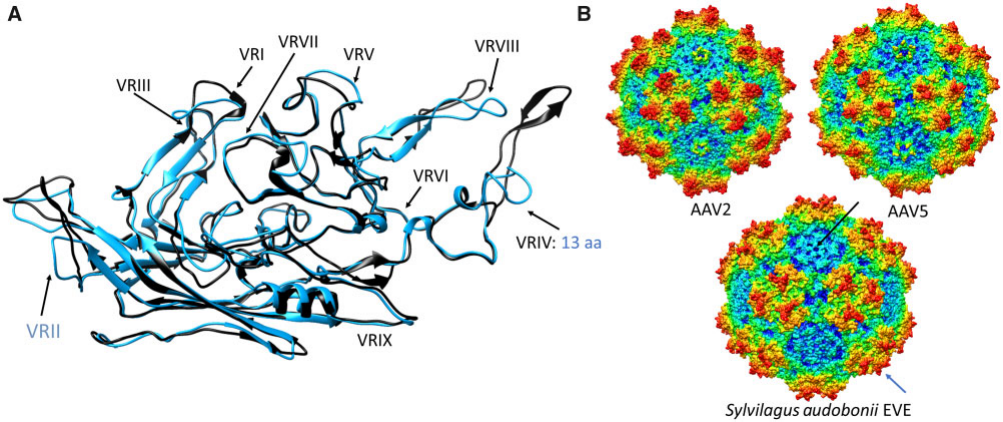
Homology modelling of the capsid proteins reconstructed from the endogenous dependoparvovirus-like elements found in members of family Leporidae of order Lagomorpha, represented by the most conserved such element derived from *S.audobonii*. (A) Ribbon diagram of the *S.audobonii* capsid monomer homology model (blue) superimposed on the monomer of the AAV2 capsid (black). The blue letters indicate deletions, specifying the number of aa or the region affected, compared to the reference structure of AAV2. As the entire VRII is absent, this is highlighted in blue. (B) The complete capsid homology model of *S.audobonii*, with the AAV2 and 5 capsids provided for comparison. The black arrow points to the DE loop, which is missing the entire VR-II. The blue arrow highlights the truncated threefold protrusions, caused by the deletion affecting VRIV.

**Figure 4.**
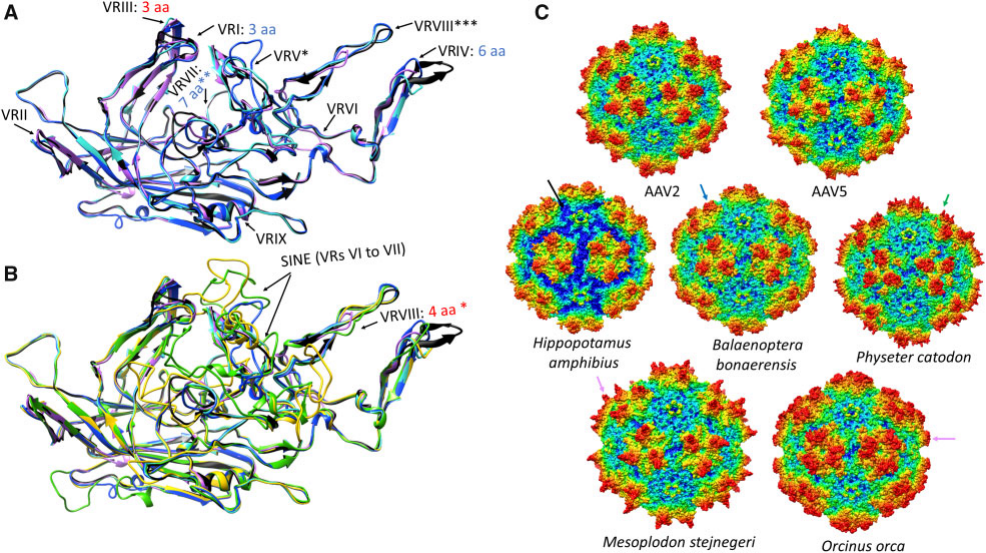
Homology modelling of the capsid proteins reconstructed from the endogenous dependoparvovirus-like elements found in members of Whippomorpha. Each capsid variation is represented by one whippomorph species. VR stands for variable region. (A) Ribbon diagram superposition of the models of the most intact capsid protein monomers of the hippopotamus (pink ribbon), a baleen whale (cyan ribbon) and a basal toothed whale (blue ribbon), none of them affected by the SINE insertion. Insertions are indicated by red text, deletions by blue letters, specifying the number of aa affected, compared to AAV2, which is used as the reference structure (black ribbon). Features marked by stars are as follows: *—Mutated beyond recognition in *P.catodon* EVE, though present; **—Not affected in *H.amphibius* and *B.bonaerensis*; ***Distorted in *H.amphibius* due to the absence of the C-term. (B) Superposition of models of capsid monomers affected by the SINE insertion with those, which were not. Ribbon colors are shown as in panel A, with the addition of the *O.orca* (yellow ribbon) and the *M.stejnegeri* (green ribbon) elements. *—The four-aa-long insertion is only present in *M.stejnegeri*. (C) Complete capsid homology models of all the variations from panels A and B. The deleted C-terminal region of the *Hippopotamus amphibious* capsid model is marked by the black arrow. The blue arrow indicates the truncated threefold protrusions of the baleen whale entry, probably the closest to what could be find on the original capsid responsible for these endogenous elements. This region is heavily mutated in the basal toothwhale species, the sperm whale (*P.catodon*), marked by the green arrow. The magenta arrow shows how the SINE insertion distorts the threefold of the beaked whale and dolphin endogenous element capsids.

**Figure 5.**
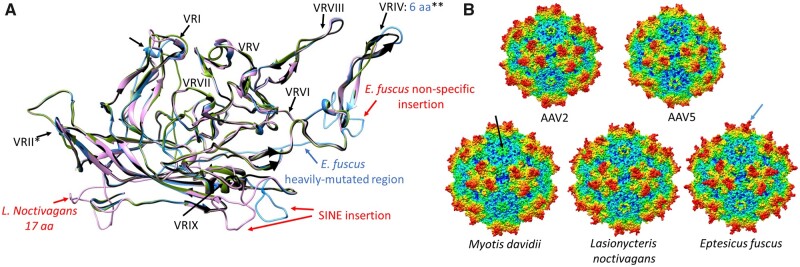
Homology modelling of the capsid proteins reconstructed from the endogenous dependoparvovirus-like elements found in members of family Vespertilionidae. Each genus is represented by one entry, except for *Plecotus*, as this capsid protein did not significantly differ from that of any of the variations found in the other three genera. (A) Ribbon diagram superimposition of the capsid protein monomer models of the *L.noctivagans* (pink), *Eptesicus fuscus* (blue) and the *M.davidii* (green) elements on the AAV2 capsid monomer ribbon diagram (black). Insertions are indicated by red, deletions by blue letters, specifying the number of aa affected, compared to the reference structure of AAV2. The small hollow arrows indicate structural changes due to the missing βG sheet of the jelly roll in genus *Myotis*. The following features are indicated by the stars are as follows: *—A deletion of four aa in the surrounding region of genus *Myotis*; **—the *E.fuscus* element is an exception. (B) Complete capsid homology models of all the variations from section A. The black arrow points to the DE loop of *M.davidii*, which results in a significantly truncated fivefold channel due to the two-aa-long deletion pre- and another two within the VRII. The AAV2-like threefold peaks of *E.fuscus* are highlighted by the blue arrow.

Sequences and models for VP showed the entire VRII of Dp.3-Lagomorpha was absent, probably due to a deletion (Fig. 3). This exposed surface region is located at the peak of the DE-loop located between the βD and βE strands and five of which form the top of the channel at the capsid fivefold axis of symmetry. The deletion significantly shortens the fivefold channel (Fig. 3). The equivalence of VRIV contains four to seven aa deletions in all EPVs, leading to a model with threefold protrusions that are less pronounced than the type member, AAV2, and more like the rounder AAV5 capsid (i.e. less spikey) (Fig. 3). For Dp.1-Whippomorpha, despite the VRVI and VRVII remaining relatively unaltered in some species, the models showed differences in beaked whale and dolphin EVEs due to SINE integrations (Fig. 4). Although most Leporidae and Whippomorpha EVE VRs remained consistent within orthologues, VRs differed for orthologous sequences of vespertilionidae species possessing Dp.2-integrations (Supplementary Table S4 and Fig. 5). Variability of the VP1 aa sequences between orthologues differed amongst the three mammalian taxa. Despite having the earliest integration time and most disrupted nature, the Dp.3-Leporidae VP1 orthologues proved to be the most conserved, incorporating the least amount of mutations (Supplementary Fig. S6).

## 3. Discussion

**Figure 6.**
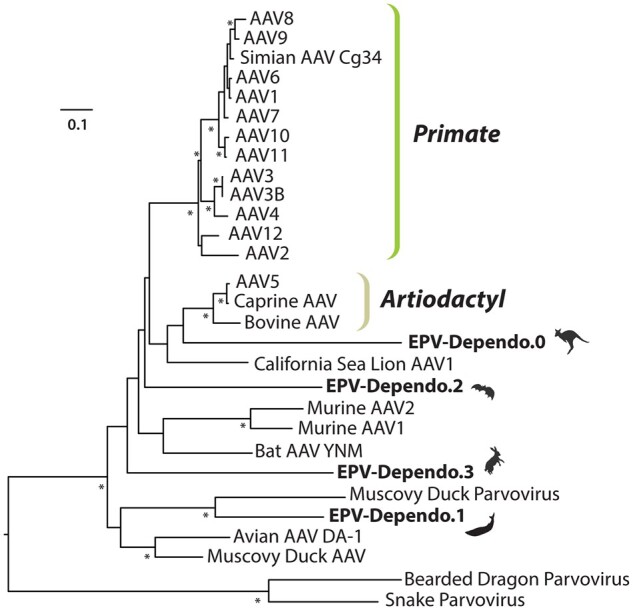
Maximum likelihood phylogenetic tree of circulating dependoparvoviruses and EVEs based on alignment of NS protein sequences using RAxML and 1,000 bootstrapping replicates. EVEs are indicated in bold with images of host species after their name.

EVEs represent genomic fossils that function as surrogates for ancestral viral genomes able to provide insight into ancient, presumed extinct, viruses. These EPVs derived from ancestral dependoparvoviruses represent distinct viral genotypes, including the three distinct orthologues of this study, confirmed by their independent clustering relative to extant AAVs in phylogenetic trees (Fig. 6). We were able to determine an estimated minimum age for their integrated viruses. The Cetacean and Lagomorph integrations appear to be older events, both occurring within a similar geologic timespan for these lineages. EVEs from Vespertilionidae bats and another previously reported, full-length AAV–EVE in Macropodidae (Smith et al. 2016), are relatively younger. Interestingly, both of these integration intervals overlap with two large adaptive radiation events of mammals; one that directly followed the Cretaceous–Paleogene mass extinction event, approximately 65 MYA, and one after the Oligocene–Eocene extinction, about 33 MYA. These extinction events are likely to have influenced the diversity and evolution of host-specific DNA viruses infecting mammals, by either conquering novel and rapidly emerging niches in the form of newly diverged host species or co-divergence, which would imply that dependoparvovirus radiation strictly followed their hosts. Nonetheless, our results corroborate with previous findings suggesting genus *Dependoparvovirus* to be at least 50 million years old (18).

### 3.1 Distinct endogenous dependoparvoviral element lineages display genome organization of both typical and unique features for genus *Dependoparvovirus*, shedding light on intragenic viral evolution

The EPV identified from whippomorph genomes groups as a sister clade to waterfowl dependoparvoviruses and shares the highest Rep aa identity percentage with them (Fig. 6 and Table 1). Members of species *Anseriform dependoparvovirus 1* possess characteristics distinguishing them from mammalian members of genus *Dependoparvovirus*, namely their pathogenicity in waterfowl and autonomous replication capabilities (Zádori et al. 1995). In addition, waterfowl dependoparvoviruses harbour a distinct transcription strategy with an alternative extra intron in mRNAs and absence of the AAV P19. Instead they translate their small Rep proteins from a short 5′ exon that originates from an ORF distinct from *rep* via alternative splicing (Qiu et al. 2005). No homologous promoter candidate for P19 could be identified in the whippomorph EVEs despite good preservation of the P49 homologue, further suggesting shared characteristics between modern avian dependoparvoviruses and ancient whippomorph EPVs.

The *Dependoparvovirus* genus is hypothesized to have a common avian–reptilian (Diapsida) origin, based on both the possibility of autonomous replication in waterfowl and squamate reptiles, as well as via phylogenetic inference (Farkas et al. 2004; Pénzes and Benkő 2014; Pénzes et al. 2015). Our results suggest that EPVs might help point specifically to squamate reptiles only, as dependoparvoviruses infecting extant snakes and lizards, despite existence of these ancient EPVs, still comprise the stemgroup of the genus. Ancient dependoparvoviruses may have diverged from reptilian ancestors to both birds and mammals on at least two separate occasions, consistent with two, rather divergent lineages of derived dependoparvoviruses. Our results suggest the possibility that ancient mammalian dependoparvoviruses, of which the whippomorph element represents, may have had the capability of autonomous replication and pathogenicity, similarly to waterfowl dependoparvoviruses clustering in the same clade as Dp.1. However, to investigate these proposed phenotypes isolation and characterization of extant exogenous cetacean dependoparvoviruses would be necessary.

EVEs from Lagomorpha and Chiroptera species both cluster within the third large dependoparvoviral lineage that infects both avian and mammalian hosts (Fig. 6). This may indicate that the aforementioned radiation might have happened multiple times, preserving elements of the ancestral, reptilian dependoparvoviral genome organization, with respect to *rep* transcription. This was confirmed in both the Vespertilionidae and leporid EVEs, as all possess three promoters and possibly the short 3′ encoding exon of Rep68 and Rep40.

While there are no known exogenous dependoparvoviruses for Lagomorpha and Whippomorpha species, Chiropteran AAVs have been identified from a variety of bat species, including a fully sequenced genome from *Myotis ricketti* (Li et al. 2010). Despite the presence of both endogenous and exogenous AAVs within *Myotis* bats, the vesper bat EVE clustered outside of the clade containing modern circulating *Myotis* bat-derived dependoparvoviruses (Fig. 6). This strongly suggests that dependoparvovirus radiation among bats has occurred independently at least twice and that distinct dependoparvovirus types, possibly even species, could infect the same host (i.e. *Myotis*).

### 3.2 Conservation bias between rep and cap; family Leporidae harbours dependoparvovirus EVEs with intact, potentially expressible Rep and suggests large Rep N-term flexibility

Despite existing for millions of years within host genomes, EVEs are still recognizable as remnants of AAV genomes. This suggests that evolutionary pressures are not driving the host cell to eliminate or significantly mutate this exogenous DNA via purifying selection after integration into host genomes. EVE transcripts were detected in the transcriptome of lagomorph and cetacean species. Other parvovirus-derived EVEs have been previously identified in South American rodents and African elephants (*Loxodonta africana*) that also showed expression of EPVs and harboured intact NS- and Rep-encoding genes (Arriagada and Gifford 2014; Kobayashi et al. 2019). Based on these examples of multiple EVEs conserved across diverse mammalian taxa that have maintained expression in the host transcriptome suggests conservation of EVE sequences, and even expression, may confer a selective advantage to the host or provide a co-opted function of original parvoviral genes. Previously, domestication and co-option of EVEs have been shown to undergo positive selective pressures and impart novel functions for immune regulation and transcriptional response in retroviruses (Arnaud et al. 2007; Dewannieux and Heidmann 2013; Chuong, Elde, and Feschotte 2016). Therefore, it is feasible that similar mechanisms could be a factor affecting the role of AAV–EVE integrations, providing possible explanations for the conservation of dependoparval-EVEs through multiple speciation events within these lineages. For example, in the case of an EVE of amdoparvovirus origin identified within the Transcaucasian mole vole (*Ellobius lutescens*) genome, an intact NS gene fused with a bZIP transcription factor’s ORF (Pénzes et al. 2018). This fusion suggests mutual expression of the viral NS with the transcription factor, which would illustrate an example of parvovirus EVEs that echoes similar findings from endogenous retroviral integrations shown to have become co-opted as transcription-modulating sequences within the host (Chuong, Elde, and Feschotte 2016).

Peculiarly, the age of the EPVs did not correlate with the degree of *rep* preservation: among the chiropteran, whippomorph, and lagomorph EPVs the Leporidae *rep* was the most intact, despite its antiquity. Moreover, this EVE proved to be transcriptionally the most active, with transcripts spanning the entire ancient *rep* ORF, supporting modelling predictions that the well-preserved EPV promoters are likely functional in the rabbit cell line. Despite its capability for transcription, the Leporidae rep ORF harbours the most divergent protein sequence out of the three elements. This suggests that the ancestral Leporidae dependoparvovirus might have displayed similar, relatively low, sequence conservation with mammalian and avian dependoparvoviruses already, an unusual phenomenon in this otherwise highly conservative *Parvoviridae* genus. Interestingly, this was not ubiquitous throughout the entire Lagomorpha order, as the Ochotonidae family had almost completely lost the entire homologous EVE. This suggests that serendipity might also play a role in these events, as well as other intra- and extracellular factors responsible for shaping the host cell fitness landscape. Studies examining primate AAV integration events demonstrated that the epigenetic status of the host cell genome significantly influences integration efficiency as well as the efficiency of gene expression (Chanda et al. 2017). Therefore, differences in host cell epigenetic status of ancestral lagomorphs during divergence of Ochotonidae and Leporidae families could be one component impacting fixation differences in this order.

Although the occurrence of transcription does not necessarily imply mRNA translation, the leporid EVE is the only EPV in this study that could potentially express Rep proteins, namely Rep52/40. The p40 Rep proteins possess non-processive, monomeric, 3′-5′ helicase functions, with constitutive ATPase activity that might facilitate cellular DNA replication, explaining one possible cellular benefit of co-opting them (Collaco et al. 2003). The ancestral sequence, however, possesses two possible reading frames encoding the N-terminal region of the large Reps. The N-terminal region of Rep78/68 is responsible for DNA-binding activity (Chiorini et al. 1994), nickase activity (Im and Muzyczka 1990), and ligase activity (Smith and Kotin 2000), as well as for activation of both viral and heterologous promoters for regulating gene expression in the host cell (Weger et al. 1999). Homology modelling suggests that the exogenous counterpart of this EVE might have harboured two variations of its large Reps. Modelling also predicts structural differences between ORF A and B, suggesting that there might have been different functions associated with these Reps.

BLASTx searches of proteomic databases available for *O.cuniculus* predict a potential NS protein based on the EPV sequence (PREDICTED: uncharacterized protein LOC103349423, NCBI Reference Sequence: XP_008259747.1). Regardless of translation, the illustrated transcription of Cetacean and Leporidae EPV sequences may provide a direct benefit for the hosts themselves, particularly as non-coding RNA (ncRNA). Specifically, ncRNAs have been identified that are important components for regulating innate immune responses and stimulating antiviral responses (Nishitsuji et al. 2016; Qiu et al. 2018). Endogenous retrovirus elements have been identified that encode ncRNAs able to activate immune stimulating genes to trigger innate immunity pathways and inhibit entry and replication of exogenous viruses (Chen et al. 2019). This mechanism could provide another selective advantage as disease resistance, which might explain even the cetacean and Leporidae EVEs.

Considering that AAV integration into host genomes is an established phenomenon during both natural infections and gene therapy administration (Kotin et al. 1990; Kaiser 2020), it appears that relatively few integrations become EVEs passed on in germline tissues. Particularly for humans, which have a preferential AAV integration site known as the AAVS1 locus, there are no known human AAV–EVEs reported thus far (Smith et al. 2016). Collectively this suggests that the acquisition and fixation of dependoparvoviral EVEs is likely affected by multiple factors beyond simple integration frequency.

### 3.3 Reconstructed 70 to 27 million-year-old capsid proteins of dependoparvovirus EVEs reveal dynamic PLA2 domains and VRs, likely reflecting pre-integration adaptations

Homology modelling of these ancestral capsids revealed that despite the estimated age and disrupted nature of viral sequences, template recognition reliably identifies reconstructed VP protein sequences to be of dependoparvoviral origin. Capsid models show striking resemblance to extant dependoviral capsids, namely prominent, separated threefold protrusions compared to other members of the family (Chapman and Agbandje-Mckenna 2006; Tijssen et al. 2011). Both the Vespertilionidae and the cetacean EVE VPs show remarkable variance, especially at VRs. Although VRs provide the surface loops of the exogenous virus capsid, which interact with components of host cells essential for reaching the nucleus for replication or egress, no such function was necessary after integration, unless these *cap* ORFs were still under purifying selection post integration. The reason why VRs accumulate mutations at a greater rate than the constant VP coding regions even after integration is likely because these were originally required to establish a niche in susceptible hosts.

In contrast, deletions affecting the Leporidae EVE VRs are consistent throughout all eight species, suggesting these mutations may reflect true diversity present in the viral genome pre-integration and not random mutations occurring within individual host species post-integration. One such deletion renders the entire variable stretch of the DE loop absent, significantly truncating the fivefold channel. Although it has been demonstrated empirically that mutations affecting the HI loop give rise to assembly-deficient mutants, no such studies have been conducted on the DE loop (DiPrimio et al. 2008). Interestingly, the length of this channel varies greatly throughout the *Parvoviridae* family, suggesting this area to be evolutionally dynamic (Tijssen et al. 2011). Deletion of the VRII also results in loss of the basic region of the AAP, which is one of the essential functional regions of this protein (Tse et al. 2018). If this deletion occurred pre-integration, capsid assembly must have been carried out efficiently without the scaffolding function of AAP, as has been reported for extant AAV4, 5, and 11 serotypes (Earley et al. 2017). The length and topology of the other affected VR, VRIV, is the one of the most variable throughout the *Dependoparvovirus* genus. As one of the regions forming the threefold protrusions, it is associated with antibody, as well as with receptor binding (Raupp et al. 2012; Tseng et al. 2015; Huang et al. 2016). Differences in VRIV of the leporidae EVEs also included deletions. The ubiquitous nature of both VRII and VRIV deletions, especially in contrast to EVEs of the other two mammalian orders, suggests these mutations occurred pre-integration, representing true adaptations of the ancient exogenous virus capsid.

The PLA2 domain, which is essential for parvovirus infection, has also undergone significant changes compared to extant dependoparvoviruses. All EVEs displayed intact, well-preserved Ca^2+^-binding loops but not conserved catalytic regions, in which deletions were observed. Recently identified amdoparvovirus and protoparvovirus-like EVEs displayed similar deletions in the same region of their PLA2 (Pénzes et al. 2018). In addition, there are two exogenous parvovirus genera (*Avi*- and *Amdoparvovirus*), which lack this conserved domain (Tijssen et al. 2011; Cotmore et al. 2014). These findings indicate that the loss of the PLA2 domain occurred on multiple occasions in subfamily *Parvovirinae* and EVEs without functional catalytic regions could represent transitional forms to this state. This is also supported by the ubiquitous nature of the lengthy deletion in the lagomorph EVE PLA2, suggesting its possible pre-integration existence. This EVE has also a shortened fivefold channel, which might be associated with the mutations rendering the PLA2 catalytic domain non-functional. Furthermore, homology modelling of Aleutian mink diseases virus of genus *Amdoparvovirus* also suggests a similarly shortened fivefold channel compared to that of the closely related protoparvoviruses (Pénzes et al. 2018).

### 3.4 EVEs and gene therapy; a novel approach to overcome immunogenicity limitations in patients of AAV seropositivity

EVEs provide an unexplored diversity of dependoparvoviruses, which are either unknown or missing from extant viromes. These viruses could serve as a source for novel capsids with potential use in AAV gene therapy applications. Specifically, individual candidate targets identified via homology modelling of ancestral AAV sequences indicates that major variations of EVEs primarily affect surface features of capsids. The truncated threefold VRIV seen in the Leporidae EVE changes the antibody-binding region of the capsid, thus this loop modification could be a candidate for incorporation into novel engineered AAV vectors. Moreover, the threefold protrusions of AAVs contain glycan-interacting sites, hence this VR-IV variation might also alter tissue tropism (Huang, Halder, and Agbandje-McKenna 2014). In addition, the AAV2-like filled-in VRI, as well as the insertions and deletions of the VRIV of the Vespertilionidae EVEs, are likely of similar interest for particular genotypes that may be incorporated into capsids to improved tissue targeting or therapeutic efficiency compared to current vectors.

Previous studies have shown that AAV capsids can be engineered from a consensus viral genome based on modern circulating primate AAVs and that these ‘ancestral ANC’ capsids provide unique properties with potential utility as gene therapy vectors (Zinn et al. 2015). This provides proof-of-concept for the functional utility of an evolution-based perspective in vector design. While generation of ANC capsids utilized more recent, modern exogenous viruses from primates instead of ancient EVE sequences for inference, still, both methods consider the evolutionary history of AAV species as viable approaches for capsid design. Inclusion of EVE sequences to regenerate ancient viral genotypes increases the breadth of AAV capsid diversity beyond known primate AAVs and from a much greater timescale than reflected by modern viruses alone. These ancient AAV–EVEs represent extinct virus species that existed millions of years ago. As a result, these genotypes may provide a rich source for diverse capsids based on viral strains that have no prior exposure in human populations today. Such limited exposure would equate to low neutralizing antibody titres against EVE-based vectors, thereby increasing the pool of eligible patients for gene therapy. This would alleviate therapeutic limitations due to host immune responses resulting from prior exposure to modern AAV strains, which is a confounding factor in successful gene therapy applications that excludes many candidate patients from initial use of current AAV vectors (Mingozzi and High 2013). Therefore, EVEs offer a novel biological reservoir of AAV sequences to provide a rational basis for capsid engineering by sampling the expanded genetic diversity observed in EVEs. This allows an alternative approach utilizing evolutionary history to augment the common practice of generating combinatorial capsid libraries via random statistical permutations and incorporate evolutionary perspectives into capsid development and gene therapy.

## 4. Materials and methods

### 4.1 Samples and sequence analysis

DNA samples from forty individual cetacean, fifteen lagomorph, and ten chiropteran specimens were obtained either from cell lines (ATCC, San Diego Global, and a generous gift from Christopher F. Basler, Georgia State University), or tissue samples provided through museum collection archives (Museum of Southwestern Biology, MSB) (Loan authorization request No. 2016.020 Mamm), the University of Alaska Museum (UAM) Museum of the North (Loan authorization request No. 2013.13 Mamm), National Oceanography and Atmospheric Administration (NOAA), and the National Marine Mammals Fisheries Service (NMFS) Regional Stranding Network Associates (in cooperation with the Mystic Aquarium Stranding Network, and NMFS Regional Stranding Network (Authorization Permit No. 19768)).

DNA was extracted using Qiagen DNeasy Blood & Tissue Kit (Cat No./ID: 69506), followed by PCR amplification with Phusion High-fidelity polymerase (New England Biolabs) using primers indicated in Supplementary Table S5. Additional species with reference genomes from the NCBI were screened *in silico* for the EVE sequence and, for EVE positive species, their sequence data included with sequences derived from the tissue samples to survey a total of 19 Cetacean and one Hippo species, all members of the whippomorph suborder (Supplementary Table S1), eight lagomorph species (Supplementary Table S3), and twenty-four chiroptera species (Supplementary Table S2). DNA sequences for each species’ EVE were aligned via MAFFT (Katoh et al. 2002), then inspected and analysed for manual adjustments of alignments using Geneious v9.1.5 (Kearse et al. 2012). Maximum likelihood trees of EVE-positive species from Whippomorpha, Lagomorpha, and Chiroptera were constructed using RAxML for 500 bootstrapping replicates (Stamatakis 2014). Phylogenetic trees were generated for each taxon based on three different sets of DNA-sequence data using: 1, EVE sequences, 2, A control set of conventional gene sequences traditionally used in phylogenetic studies (mitochondrial Cytb, Actin intron, and vwf), and 3, combined control genes plus EVE sequences (Supplementary Figs S7–S9A–C).

### 4.2 Estimating age of EVE integration

Time-calibrated phylogenies for evolution and speciation of host species previously published for cetacean (McGowen, Spaulding, and Gatesy 2009), lagomorph (Matthee et al. 2004), and chiropteran (Miller-Butterworth et al. 2007; Agnarsson et al. 2011) orders were compared to phylogenetic trees utilizing EVE-positive species (Supplementary Figs S7–S9) to estimate a time range for integration of the EVE into the MRCA of the orders. Using the estimated ages for branches and nodes of host species, we determined at which points integration and fixation of the EVE in the host lineages occurred to approximate time-periods for integration and establish a relative age for the various EVE viruses.

### 4.3 Endogenous EVE sequences versus modern circulating viruses

EVE sequences previously identified within marsupial species (Smith et al. 2016) were analysed in addition to EVE sequences from the Vespertilionidae, Lagomorpha, and Whippomorpha taxa to generate consensus EVE sequences for each of the four mammalian lineages. Translated NS sequences created based on a consensus alignment of each of the four EVEs were used to align with NS sequences of modern, circulating dependoparvoviruses (Supplementary Table S6) and evaluate sequence similarity between ancestral EVE viruses and modern circulating viruses. To illustrate relationships among EVEs and extant dependoparvoviruses, this data was used to generate a phylogenetic tree containing circulating viruses and EVE sequences via RAxML (Stamatakis 2014) with 1,000 bootstrapping replicates (Fig. 6).

### 4.4 Reconstructing ancient reading frames and annotation

To examine EVEs at the protein level and gain insight into their ancestral genome organization, the ancestral reading frames for *rep*, *cap*, and AAP were reconstructed. Annotation was carried out using the Artemis Genome Browser (Carver et al. 2012). The validity of suspected original frame fragments was first evaluated by BLAST X and P algorithms. Alignment of EVEs with homologous aa sequences of dependoparvovirus protein sequences from NCBI utilizing MUSCLE and the T-Coffee suite of programmes (M-Coffee, T-Coffee, and Expresso) helped further incorporate structural data and ensure reliable detection of synonymous sites (Altschul et al. 1997; Edgar 2004; Armougom et al. 2006). Promoters were predicted using the neural network-based promoter prediction server of the Berkeley Drosophila Genome Project, as well as the Promoter Prediction 2.0 server (Knudsen 1999; Reese 2001). Predicted promoter sequences were aligned with established dependoparvoviral promoters to confirm validity. Splice sites were also detected using the neural network-based applications of the Berkeley Drosophila Genome project and SplicePort (Reese et al. 1997; Dogan et al. 2007). To verify that these applications are capable of detecting dependoparvovirus splice sites, we tested their performance by running dependoparvovirus genomes with experimentally confirmed splice sites through the workflow pipeline to validate functionality of the programmes.

### 4.5 Expression of EVE sequences within host species transcriptomes

Cell lines from four, EVE-positive species (Supplementary Table S7) were cultured in 10 per cent FBS media (Eagle’s Minimal Essential Medium or Dulbecco’s Modified Eagle Medium, as recommended), then trypsinized and total RNA extracted via a Qiagen RNeasy Mini kit (Qiagen Cat No./ID: 74104), followed by cleanup with Invitrogen’s DNA-free Kit (No. 00555680) to eliminate residual genomic DNA contamination. RNA integrity was assessed using an Advanced Analytics Fragment Analyzer prior to submission for RNA sequencing (Genewiz, South Plainfield, NJ) on the Illumina HiSeq platform (150 bp, paired-end sequencing). *Oryctolagus cuniculus* and *T.truncatus* reads were mapped to their available reference genomes (OryCun2.0 Assembly GCA_000003625.1 or NIST Tur_tru v1 Assembly GCF_001922835.1). RNA-seq reads from species lacking a complete reference genome (*B.physalus* and *M.velifer incautus*), were mapped to their respective EVE sequences determined via Sanger sequencing.

### 4.6 Homology modelling

As homology modelling does not tolerate unknown or missing residues, it was necessary to reconstruct the presumed original residues altered by in-frame stop codons prior to further analysis. Alignments were done, with or without the incorporation of structural data as described above, to evaluate stop codons based on a parsimonic evolutionary approach to reconstruct the most-probable original codon. Briefly, we utilized a substitution matrix, in which transition and transversion events of aa codons received weighted scores based on the expected probabilities for the different types of substitutions, assuming transitions to happen at a higher frequency. For each stop codon position all potential residues observed in exogenous AAV data were considered as candidates. Potential residues in which the least number of nucleotide substitutions required to restore a coding residue, according to the scoring matrix, were selected to replace the stop codons in question. The EVE from *Sylvilagus audobonni* displayed the most intact capsid, therefore this sequence was selected for protein modelling building for Leporidae, whereas one representative of all capsid variations was subjected to model building from the other two taxa.

To identify template protein structures to guide structural modelling of EVE capsids, the pGenTHREADER and pDomTHREADER algorithms of the PSIPRED Protein Sequence Analysis Workbench were utilized (Lobley, Sadowski, and Jones 2009). This indicated that VP3 of AAV5 (RCSB PDB No. 3NTT) in case of the whippomorph EVE, the VP3 of AAV9 (RCSB PDB No. 3UX1) for the Leporidae, and the VP3 of AAV3B (RCSB PDB No. 3KIC) for the vespertilionid EVE were the most suitable protein structure templates to guide modelling of each of the respective EVEs. Homology modelling was carried out by SWISS-MODEL using the above mentioned PDB structures as templates (Biasini et al. 2014). 60mer capsids of the acquired capsid monomer models were constructed by the Oligomer Generator subroutine of the Viper web database (http://viperdb.scripps.edu/) (Carrillo-Tripp et al. 2009). The UCSF Chimera application was used to visualize the models and generated figures (Pettersen et al. 2004).

## Data availability

Newly described EVE sequence data available at NCBI with GenBank accession numbers MN782247–MN782272.

## Supplementary Material

veaa043_Supplementary_Data
