# Supplementary material for: Evolution of dependoparvoviruses across geological timescales—implications for design of AAV-based gene therapy vectors
Source: Virus Evol. 2020 May 22;6(2):veaa043. doi: 10.1093/ve/veaa043 (PMC7474932; doi:10.1093/ve/veaa043)
Supplement: veaa043_Supplementary_Data [file ve_6_2_veaa043_s7.zip › FigS3 convert.pdf]

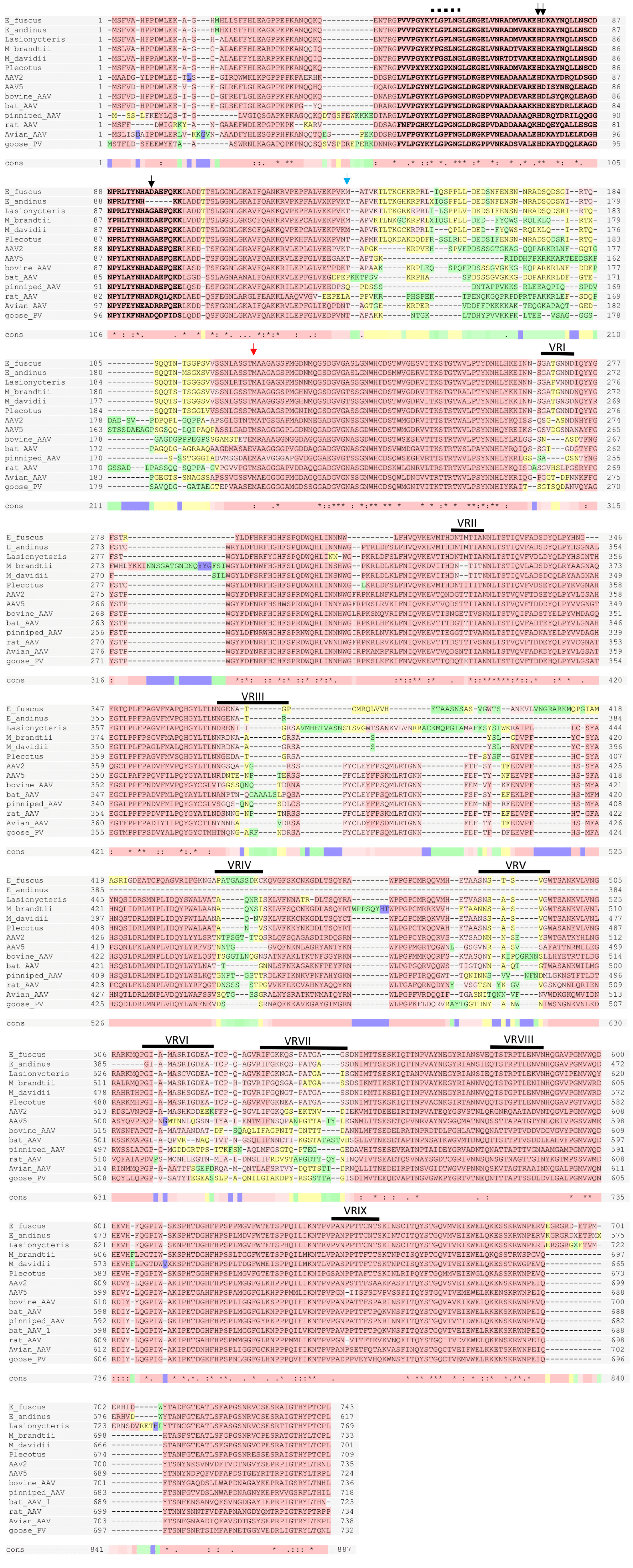

E_fuscus
E_andinus
Lasionycteris
M_brandtii
M_davidii
Plecotus
AAV2
AAV5
bovine_AAV
bat_AAV
pinniped_AAV
rat_AAV
Avian_AAV
goose_PV
bat_AAV_1
cons
VRI
VRII
VRIII
VRIV
VRV
VRVI
VRVII
VRVIII
VRIX
