# Supplementary material for: Evolution of dependoparvoviruses across geological timescales—implications for design of AAV-based gene therapy vectors
Source: Virus Evol. 2020 May 22;6(2):veaa043. doi: 10.1093/ve/veaa043 (PMC7474932; doi:10.1093/ve/veaa043)
Supplement: veaa043_Supplementary_Data [file ve_6_2_veaa043_s7.zip › FigS4.pdf]

A

```
AAAV          MR----------SYYEVIVQLPNDVESQVPGISDSFVNWITSREWTLPEDADWDLDQVDQVQLTLGDKIQREIRTHWGTMAK-EPDF
AAV1          MP----------GFYEIVIKVPSDLDEHLPGISDSFVSWVAEKEWELPPDSDMDLNLIEQAPLTVAEKLQRDFLVQWRRVSK-APEA
AAV2          MP----------GFYEIVIKVPSDLDEHLPGISDSFVNWVAEKEWELPPDSDMDLNLIEQAPLTVAEKLQRDFLTEWRRVSK-APEA
AAV3          MP----------GFYEIVLKVPSDLDERLPGISNSFVNWVAEKEWDVPPDSDMDPNLIEQAPLTVAEKLQREFLVEWRRVSK-APEA
AAV4          MP----------GFYEIVLKVPSDLDEHLPGISDSFVSWVAEKEWELPPDSDMDLNLIEQAPLTVAEKLQREFLVEWRRVSK-APEA
AAV5          MA----------TFYEVIVRVPFDVEEHLPGISDSFVDWVTGQIWELPPESDLNLTLVEQPQLTVADRIRRVFLYEWNKFSK-Q-ES
Brachylagus   ------------------------------------LSWTGSPRRMDSEDSDMDLSQSEQPRLTITESSQG-ISEGMQKVNE-IQPK
Lepus_am      MA----------GVYAANVRIPSDADC--TSSWDSRLSWTGSPRRMDSEDSDMDLSQIEQPRLTITPIGSQGISEGMQKVNE-IQPK
Oryctogalus   ------------------------------------LSWTGWPRRMDSEDSDMDLSQIEQPRLTITESSQG-ISEGMQKVNE-IQPK
S_audobonii   ------------------------------------LSWTGSPRRMDSEDSDMDLSQIEQSRLTITEKSSQGLSEGMQKVNE-IQPK
S_floridanus  ------------------------------------LSWTGSPRRMDSEDSDMDLSQIEQPRLTITESSQG-TSEGMQKVNE-IQPK
bat           ME-----------FYSIVLRLPGDFDSEVPGLQDSFYKWISGPRRELPEWSDLDPGQIESAYQILADKLVREFAQKWAAFSE-DPRA
bovineAAV     MA----------TFYEVIVRVPFDVEEHLPGISDNFVDWVTGQIWELPPESDLNLTLIEQPQLTVADRIRRVFLYEWNKFSK-Q-ES
caprine       MA----------TFYEVIVRVPFDVEEHLPGISDSFVDWVTGQIWELPPESDLNLTLIEQPQLTVADRIRRVFLYEWNKFSK-Q-ES
duck          MAFSRPLQISSDKFYEVIIRLPSDIDQDVPGLSLNFVEWLSTGVWE--PTGIWNMEHVNLPMVTLADKIKNIFIQRWNQFNQ-D-ET
sealion       MA----------SFYEVVAKIPSDLEEHLPGICDDFVSVIMSKEWRLPESSDLVLDQIDQPKLTIADAVRKVFYFEWLKYCRDVEEP
simian        MP----------GFYEIVIKVPSDLDEHLPGISDSFVNWVAEKEWELPPDSDMDLNLIEQAPLTVAEKLQRDFLVKWRRVSK-APEA

cons                                               .            .       :     :                .

AAAV          HYFIQLEQGEVFFHLHVLLETCSVKPMVLGRYIRHIQQKIVSKVYCATSLRWKDGCVVTKTKN-FGGANKVRAESYIPAYLIPKQQP
AAV1          LFFVQFEKGESYFHLHILVETTGVKSMVLGRFLSQIRDKLVQTIYRGIEPTLPNWFAVTKTRNGAGGGNKVVDECYIPNYLLPKTQP
AAV2          LFFVQFEKGESYFHMHVLVETTGVKSMVLGRFLSQIREKLIQRIYRGIEPTLPNWFAVTKTRNGAGGGNKVVDECYIPNYLLPKTQP
AAV3          LFFVQFEKGETYFHLHVLIETIGVKSMVVGRYVSQIKEKLVTRIYRGVEPQLPNWFAVTKTRNGAGGGNKVVDDCYIPNYLLPKTQP
AAV4          LFFVQFEKGDSYFHLHILVETVGVKSMVVGRYVSQIKEKLVTRIYRGVEPQLPNWFAVTKTRNGAGGGNKVVDDCYIPNYLLPKTQP
AAV5          KFFVQFEKGSEYFHLHTLVETSGISSMVLGRYVSQIRAQLVKVVFQGIEPQINDWVAITKVKK--GGANKVVDSGYIPAYLLPKVQP
Brachylagus   LFY-TI------XTISMLLPEPXVXRARLYSIPCASNSKQTQNFRRRMSSPGFYWTAIIKNSN-LDGANQIXDQAYIPAYPLGKSQP
Lepus_am      LSY-TI------XTISTLLSEPXVXRARLYSMPCASNAKQAQNFRLRMSSPGFYWTAIIKNSN-LEGANQIQDQAYVPAYPLGKRQP
Oryctogalus   QFY-TI------XTISTLLSEPXVXRARLYSMPCASNSKQIQNFRLRMSSPGFYWMAIIKNSN-LEGANQIRDQAYIPAYPLGKSQP
S_audobonii   LFY-TI------XTISMLLPEPXVXRARLYSMPCASNSKQTQNFRRRMSSPGFYWTAIIKNSN-LEGANQIRDQAYIPAYPLGKSQP
S_floridanus  LFY-TI------XTISMLLPEPXVXRARLYSIPCASNSKQTQNFRRRMSSPGFYWTAIIKNSN-LDGANQIRDQAYIPAYPLGKSQP
bat           PYFAQLEKGRENFHVHVLASSKKVGSFVVGRYVRKMRQHLVDVVFRKCEPVDADWLQVQKSGN--HKSNEIKDEGFIPAYLLPKRQS
bovineAAV     KFFVQFEKGSEYFHLHTLVETSGISSMVLGRYVSQIRAQLVKVVFQNIEPRINDWVAITKVKK--GGANKVVDSGYIPAYLLPKVQP
caprine       KFFVQFEKGSEYFHLHTLVETSGISSMVLGRYVSQIRAQLVKVVFQGIEPQINDWVAITKVKK--GGANKVVDSGYIPAYLLPKVQP
duck          DFFFQLEEGSEYIHLHCCIAQGNVRSFVLGRYMSQIKDSILRDVYEGKQVKIPDWFSITKTKR--GGQNKTVTAAYILHYLIPKKQP
sealion       LFFFQFEKGKENFHVHMVIETSNVSSMVLGRYIGTIKKKLVRKVFREVEPQMPDWLAVTKTKQ-SGGVNKTYDKGYIPAYLLPKTQP
simian        LFFVQFEKGESYFHLHILVETTGVKSMVLGRFLSQIRDKLVQTIYRGIEPTLPNWFAVTKTRNGAGGGNKVVDECYIPNYLLPKTQP
```

```
cons            :  :        :        :    :       .      .    .        : *  .     *:     ::  * : * *.

                                     End of frame "A"
AAAV           EV------------------------QWAWTNVPEYIKACLHRELRASLARLHFEEAGVSQS-KENLARTADGAPVMPTRVSKRYME
AAV1           EL------------------------QWAWTNMEEYISACLNLAERKRLVAQHLTHVSQTQEQNKENLNPNSDAPVIRSKTSARYME
AAV2           EL------------------------QWAWTNMEQYLSACLNLTERKRLVAQHLTHVSQTQEQNKENQNPNSDAPVIRSKTSARYME
AAV3           EL------------------------QWAWTNMDQYLSACLNLAERKRLVAQHLTHVSQTQEQNKENQNPNSDAPVIRSKTSARYME
AAV4           EL------------------------QWAWTNMDQYISACLNLAERKRLVAQHLTHVSQTQEQNKENQNPNSDAPVIRSKTSARYME
AAV5           EL------------------------QWAWTNLDEYKLAALNLEERKRLVAQFLAESSQRSQ-EAASQREFSADPVIKSKTSQKYMA
Brachylagus    EL-----------------------WQWAWTNMEAYIRAALPPVERERPVEDHPTDIPSKFS--PDSF-SEDSGPVTRTQISQKYMA
Lepus_am       ELKEPIRFKIRPMFLPTLWESANQNWQWAWTNMEEYIRAALPPVERESPVEDRPTDVPSKFS--PDSF-SEDSGPVTRTRISQKYMA
Oryctogalus    ELN----------------------WQWAWTNMEEYIRAALPLVERECPVEDRPTDVPSKFS--PDSF-SEDSGPVTRTKISQKYMA
S_audobonii    EL-----------------------WQWAWTNMEAYIRAALPPVERERPVEDHPTDIPSKFP--PDSF-SEDSGPVTRTQISQKYMA
S_floridanus   EL-----------------------WQWAWTNMEAYIRAALPPVERERPVEDHPKDIPSKFS--PDSF-SEDSGPVTRTQISQKYMA
bat            EL------------------------QWAWTNIEKYERATLSVAERARLVEEWKRSLAAEES---DPA-EPERRPRKSTKSASEYMA
bovineAAV      EL------------------------QWAWTNLEEYKLAALNLEERKRLVAQFQLESSQRSQ-EASSQRDVSADPVIKSKTSQKYMA
caprine        EL------------------------QWAWTNLDEYKLAALNLEERKRLVAQFLAESSQRSQ-EAASQREFSADPVIKSKTSQKYMA
duck           EL------------------------QWAFTNMPLFTAAALCLQKRQELLDAFQESEMNAVV--QEDQ-ASTAAPLISNRAAKNYSN
sealion        EL------------------------QWAWTNIEEYKSASLNLAERKRLVDEFLASLRRDGPSQSEPDDQQPHGPVIRNRTSQKYMA
simian         EL------------------------QWAWTNMEEYISACLNLAERKRLVAQHLTHVSQTQEQNKENLNPNSDAPVIRSKTSARYME

cons           *:                        ***:**:  :  * *    *                            *   .: : .*


AAAV           LVDWLVEKGITTEKEWLLENRESFRSFQASSNSARQIKTALQGAIQEMLLTKTAEDYLVGKDPVSDDDIRQNRIYKILELNHYDPAY
AAV1           LVGWLVDRGITSEKQWIQEDQASYISFNAASNSRSQIKAALDNAGKIMALTKSAPDYLVGPAPP--ADIKTNRIYRILELNGYEPAY
AAV2           LVGWLVDKGITSEKQWIQEDQASYISFNAASNSRSQIKAALDNAGKIMSLTKTAPDYLVGQQPV--EDISSNRIYKILELNGYDPQY
AAV3           LVGWLVDRGITSEKQWIQEDQASYISFNAASNSRSQIKAALDNASKIMSLTKTAPDYLVGSNPP--EDITKNRIYQILELNGYDPQY
AAV4           LVGWLVDRGITSEKQWIQEDQASYISFNAASNSRSQIKAALDNASKIMSLTKTAPDYLVGQNPP--EDISSNRIYRILEMNGYDPQY
AAV5           LVNWLVEHGITSEKQWIQENQESYLSFNSTGNSRSQIKAALDNATKIMSLTKSAVDYLVGSSVP--EDISKNRIWQIFEMNGYDPAY
Brachylagus    VVNWHVENGTTSGKPWMRENSKSYLSFSSTKNARSQIRSALDNFTKLMGLTIMVLDCLVGVLAL--PDTANSRTHQILQMSSCHLAC
Lepus_am       VVNWHVENGTTSGKPWMRGNSKSYLSFSSTKNARSQIRSALDNFTKVMSLTIMVLDCLVGVLAL--PDTANSRTHQILQMSGCHRAC
Oryctogalus    VVNWHVENGTTSGKPWMQENSKSYLSFSSTKNARSQIRSALDNFTKVMGLTIMVLDCLVGVLAL--PDTANGRTHQILQMSGCHRAC
S_audobonii    VVNWHVENATTSGKPWMRENSKSYLSFSSTKNARSQIRSALDNFTKVMGLTIMVLDCLVGVLAL--PDTANSRTHQILQMSGCHLAC
S_floridanus   VVNWHVENGTTSGKPWMRENSKSYLSFSSTKNARSQIRSALDNFTKLMGLTIMVLDCLVGVLAL--PDTANSRTHQILQMSGCHLAC
bat            LVRWLVDNGIATEREWMREDSDGYLSYNATGATRAQIKAALDNAARIMVNTKTAADYLVGRNPP--LDVEDNRIYRLFRMNGYDPAY
bovineAAV      LVSWLVEHGITSEKQWIQENQESYLSFNSTGNSRSQIKAALDNASKIMSLTKSASDYLVGQTVP--EDISENRIWQIFDLNGYDPAY
```

```
caprine       LVNWLVEHGITSEKQWIQENQESYLSFNSTGNSRSQIKAALDNATKIMSLTKSAVDYLVGSSVP--EDISKNRIWQIFEMNGYDPAY
duck          LVDWLIEMGITSEKQWLTENKESYRSFQATSSNNRQVKAALENARAEMLLTKTATDYLIGKDPV--LDITKNRIYQILKLNNYNPQY
sealion       LVSWLVENGITSEKQWIQEDQESYLSFNAAGSSRSQIKSALDNASRIMSLTKKASDYLVGQSVP--EDITENKIYQLFKMNGYDPAY
simian        LVGWLVDRGITSEKQWIQEDQASYISFNAASNSRSQIKAALDNAGKIMALTKSAPDYLVGPSLP--VDITQNRIYRILALNGYDPAY

cons          :* * :: . :: : *:  :  .: *:.::     *:::**:.    *  *  . * *:*       *   .:  ::: :.  .


AAAV          VGSILVGWCQKKWGKRNTLWLFGHATTGKTNIAEAIAHAVPFYGCVNWTNENFPFNDCVEKMIIWWEEGKMTAK-VVETAKAILGGS
AAV1          AGSVFLGWAQKRFGKRNTIWLFGPATTGKTNIAEAIAHAVPFYGCVNWTNENFPFNDCVDKMVIWWEEGKMTAK-VVESAKAILGGS
AAV2          AASVFLGWATKKFGKRNTIWLFGPATTGKTNIAEAIAHTVPFYGCVNWTNENFPFNDCVDKMVIWWEEGKMTAK-VVESAKAILGGS
AAV3          AASVFLGWAQKKFGKRNTIWLFGPATTGKTNIAEAIAHAVPFYGCVNWTNENFPFNDCVDKMVIWWEEGKMTAK-VVESAKAILGGS
AAV4          AASVFLGWAQKKFGKRNTIWLFGPATTGKTNIAEAIAHAVPFYGCVNWTNENFPFNDCVDKMVIWWEEGKMTAK-VVESAKAILGGS
AAV5          AGSILYGWCQRSFNKRNTVWLYGPATTGKTNIAEAIAHTVPFYGCVNWTNENFPFNDCVDKMLIWWEEGKMTNK-VVESAKAILGGS
Brachylagus   AGSVWAAWCPCQYGRRNTTWLFGPVTTRRSSIAEATARAAPCSGCVKWTNEIFPFRGCVQQMTIWWEEEKRTAAVVDAAKATPGGG
Lepus_am      AGSVWAAWCPCQYGRRNTTWLSGPVTTPRSSIAGATARAAPCYGCVKWTNEIFPFRGCVEQMVTWWEVEKRTAA-VADAAKATRGGR
Oryctogalus   AGSVWAAWCPCQYGRRNTTWLFGPVTTRRSSIAEATARAAPCYGCAKWTNEIFPFRGCVEQMTIWWEEEKRTAT-VVDAVKATPRGG
S_audobonii   AGSVWAAWCPCQYGRRNTTWLFGPVTTRRSSIAEATARAAPCSGCVKWTNEIFPFRGCVQQMTIWWEEEKRTAA-VVDAAKATPGGG
S_floridanus  AGSVWAAWCPCQYGRRNTTWLFGPVTTRRSSIAEATARAAPCSGCVKWTNEIFPFRGCVQQMTIWWEEEKRTAA-VVDAAKATPRGG
bat           AGSVLLGWCRTGFGKRNTVWLFGPATTGKTNLAEAISHSVPFYGCVNWTNENFPFNDCVDKMIIWWEEGKMTSK-VVESAKAILGGS
bovineAAV     AGSVLYGWCTRAFGKRNTVWLYGPATTGKTNIAEAISHTVPFYGCVNWTNENFPFNDCVEKMLIWWEEGKMTSK-VVEPAKAILGGS
caprine       AGSILYGWCQRSFNKRNTVWLYGPATTGKTNIAEAIAHTVPFYGCVNWTNENFPFNDCVDKMLIWWEEGKMTNK-VVESAKAILGGS
duck          VGSVLCGWVKREFNKRNAIWLYGPATTGKTNIAEAIAHAVPFYGCVNWTNENFPFNDCVDKMLIWWEEGKMTNK-VVESAKAILGGS
sealion       LGSILLGWCQGRFGKRNTVWLYGPATTGKTNLAEAIAHSVPFYGCVNWTNENFPFNDCVDKMLIWWEEGKMTSK-VVESAKAILGGS
simian        AGSVFLGWAQKKFGKRNTIWLFGPATTGKTNIAEAIAHAVPFYGCVNWTNENFPFNDCVDKMVIWWEEGKMTAK-VVESAKAILGGS

cons           .*:  .*    :.:**: ** * .** ::.:* * :::.*  **.:**** ***..**::*  ***  * *   *.:..**   *


AAAV          RVRVDQKCKASVPIEPTPVI--------ITSNTN--MCYVIDGNTTTFEHKQPLEDRMFKLELLTRLPDDFG-KVTKQEVRQFFRWS
AAV1          KVRVDQKCKSSAQIDPTPVI--------VTSNTN--MCAVIDGNSTTFEHQQPLQDRMFKFELTRRLEHDFG-KVTKQEVKEFFRWA
AAV2          KVRVDQKCKSSAQIDPTPVI--------VTSNTN--MCAVIDGNSTTFEHQQPLQDRMFKFELTRRLDHDFG-KVTKQEVKDFFRWA
AAV3          KVRVDQKCKSSAQIEPTPVI--------VTSNTN--MCAVIDGNSTTFEHQQPLQDRMFEFELTRRLDHDFG-KVTKQEVKDFFRWA
AAV4          KVRVDQKCKSSAQIDPTPVI--------VTSNTN--MCAVIDGNSTTFEHQQPLQDRMFKFELTKRLEHDFG-KVTKQEVKDFFRWA
AAV5          KVRVDQKCKSSVQIDSTPVI--------VTSNTN--MCVVVDGNSTTFEHQQPLEDRMFKFELTKRLPPDFG-KITKQEVKDFFAWA
Brachylagus   KVRADQICKSSAQAEPTPAVA----VAVITGNADMGMGAVADGKPTNSQHQQRLRDQRPTFERTRRLTPGFGERVPKREAKSFFRWA
Lepus_am      KVRADRICKSSAQVEPTPAVAVAVAVAVITGNADMGMGAVADGKPTNSQHQQRLQDQRSTFERTRRLTPGFGERLPKGEAKSFFRWA
Oryctogalus   KVRADQICKSSAQVEPTPAVA----VAVITGNADMGMGAVADGKPTNSQHQQRLQDQRSTFERTRRLTPGFGERLPKREAKSFFRWV
```

```
S_audobonii   KVRADQICKSSAQAEPTPAVA----VAVITGNADMGMGAVADGKPTNSQHQQRLRDPRSTFERTRRLTPGFGERVPKREAKSFFRWA
S_floridanus  KVRADQICKSSAQAEPTPAVA----VAVITGNADMGMGAVADGKPTNSQHQQRLRDQRPTFERTRRLTPGFGERVPKREAKSFFRWA
bat           KVRVDQKCKNSQQIEPTPVI--------ITSNTN--MCEVVDGNSTTFEHRQPLEDRMFKFELTVRLQPTFG-KITKQEVREFFKWA
bovineAAV     RVRVDQKCKSSVQVDSTPVI--------ITSNTN--MCVVVDGNSTTFEHQQPLEDRMFRFELMRRLPPDFG-KITKQEVKDFFAWA
caprine       KVRVDQKCKSSVQIDSTPVI--------VTSNTN--MCVVVDGNSTTFEHQQPLEDRMFKFELTKRLPPDFG-KITKQEVKDFFAWA
duck          AVRVDQKCKGSVCIEPTPVI--------ITSNTD--MCMIVDGNSTTMEHRIPLEERMFQIVLSHKLEGNFG-KISKKEVKEFFKWA
sealion       KVRVDQKCKSSVQIDSTPVI--------ITSNTD--MCCVIDGNSTTFEHRQPLEDRMFRINLEQRLSHDFG-KITKREVREFLAWA
simian        KVRVDQKCKSSAQIDPTPVI--------VTSNTN--MCAVIDGNSTTFEHQQPLQDRMFKFELTRRLEHDFG-KVTKQEVKEFFRWA

cons           **.*: ** *   :.**.:        :*.*::  *  : **:.*. :*:  *.:    :    :*   ** ::.* *.:.*: *
```

Donor site location

```
AAAV          QDHLTPVIPEFLVRKAE--------SRKRPAPSGEG--YISPTKRPAL-----------------A----E--QQQASESA------
AAV1          QDHVTEVAHEFYVRKGG--------ANKRPAPDDAD--KSEP-KRACP-----------------S----VA-DPSTSDAE------
AAV2          KDHVVEVEHEFYVKKGG--------AKKRPAPSDAD--ISEP-KRVRE-----------------S----VA-QPSTSDAE------
AAV3          SDHVTDVAHEFYVRKGG--------AKKRPASNDAD--VSEP-KRECT-----------------S----LA-QPTTSDAE------
AAV4          SDHVTEVTHEFYVRKGG--------ARKRPAPNDAD--ISEP-KRACP-----------------S----VA-QPSTSDAE------
AAV5          KVNQVPVTHEFKVPRELAGTKGAEKSLKRPLGDVTNTSYKSLEKRARL-----------------S----F--VPETPRSSDVTVDP
Brachylagus   SDPMTDVTLEFFVGKTE--------SEGRPSPSGDG-KNRRPAEKPAP----------------------AF-EPHVPS--------
Lepus_am      SDHMTXVTLEFFVGKTE--------SEGRPAPSGDG-KNRRPAEKPVL----------------------AF-EPHVPS--------
Oryctogalus   SDPMTDVTLEFFVGKTE--------SEGRPAPSGDG-KNRRPAEKPAL----------------------AF-EPHVPS--------
S_audobonii   SNPMTDVTLEFFAGKTE--------SERRPAPSGDG-KNRRPAEKPAP----------------------AF-EPHVPS--------
S_floridanus  SDPMTDVTLEFFVGKTE--------SEGRPXPSGDG-KNRRPAEKPAP----------------------AF-EPHVPS--------
bat           ELNAVDVEYDFLVRKINQSDT--GGGVKRGAEPTKD---EPPAKRVFF-----------------Y----GA-TSEGEDVR--EGAP
bovineAAV     KVNQVPVTHEFMVPKKVAGTERAETSRKRPLDDVTNTNYKSPEKRARL-----------------S----V--VPETPRSSDVPVEP
caprine       KVNQVPVTHEFKVPRELAGTKGAEKSLKRPLGDVTNTSYKSPEKRARL-----------------S----F--VPETPRSSDVTVDP
duck          NDNLVPVVSEFKVPTNE--------QTKLTEPVPER--ANEPSEPPKIWAPPTREELEEILRASPELFASVAPLPSSPDTS------
sealion       QEYEVDVEHTFEVTKLAK-----PKVTKRSAPLSDD--YKSPAKRARL-----------------I--PDLVAEEATSSAL----AE
simian        SDHVTEVTHEFYVRKGG--------ASKRPAPDDAD--ISEP-KRACP-----------------S----VA-DPSTSDAE------

cons          .   . *   * .                              :
```

```
AAAV          ---DP--VPTRYRIKCSKHCGMDKM-LFPCQICESMN------RDINICAIHKTTD-C-KECFP-DYGDKDDVELPPCTEHNVSRCY
AAV1          --GAPVDFADRYQNKCSRHAGMLQM-LFPCKTCERMN------QNFNICFTHGTRD-C-SECFPGV---------SESQ--------
AAV2          ---ASINYADRLARGHSL---------------------------------------------------------------------
AAV3          ---APADYADRYQNKCSRHVGMNLM-LFPCKTCERMN------QISNVCFTHGQRD-C-GECFPGM---------SESQ--------
AAV4          ---APVDYADRYQNKCSRHVGMNLM-LFPCRQCERMN------QNVDICFTHGVMD-C-AECFP-V---------SESQ--------
AAV5          APLRPLNWNSRYDCKCDYHAQFDNI-SNKCDECEYLN------RGKNGCICHNVTH-C-QICHG-I---------PPWE--------
Brachylagus   -------ASGRYVSTCSEHLRLGMV-KCPLKGCECVN------TALNMCFIHRTLX-F-LRVFL-M---------KQFQTKN-----
Lepus_am      -------ASGRYVSRCSEHLRLGMV-KCPLKGCECVN------TALNMCFIHGKLGGF-LRVCF-L---------KQFQTKN-----
Oryctogalus   -------ASGRYVSPCSEHLRLGTV-KYPWKGCECVN------TALNMCFIHGKLG-C-LRVFF-L---------KQFQTKN-----
S_audobonii   -------ASGRYVSTCSEHLRLGMV-KCPLKGCECVN------TALNMCFIHRKLG-F-LRVFF-L---------KQFQTKD-----
S_floridanus  -------ASGRYVSTCSEHLRLGTV-KCPLKGCECVN------TALNMCFIHRTLG-FFLRVFL-M---------KQFQTKN-----
bat           GESDSVNFAERYVSKCSKHLSWSNM-RYPCRACERMN------ADVNVCTPHGCRD-C-PECFP-R---------PAP---------
bovineAAV     APLRPLNWSSRYECRCDYHAKFDSV-TGECDECEYLN------RGKNGCIFHNATH-C-QICHA-V---------PPWE--------
caprine       APLRPLNWNSRYDCKCDHHAQFDNI-SDKCDECEYLN------RGKNGCICHNVTH-C-QICHG-I---------PPWE--------
duck          --PKRKKTRGEYQVRCAMHSLDNSMNVFECLECERANFPEFQSLGENFCNQHGWYD-C-AFCNE-L---------KDDM--------
sealion       AEEWDLNWDRRYDCRCEAHSMSVRV-EGLCRDCEYLN------RGKNMCLVHGDTG-C-HVCHA-V---------PPW---------
simian        --GAPVDFADRYQNKCSRHAGMLQM-LFPCKTCERMN------QNFNICFTHGVRD-C-SECFPGV---------SESQ--------

cons                    .

AAAV          QCHSGELYRVTSDSDEKPAPESDEGTEPSY-APCT---IHHLMGKSHGLVTCAACRLKNSTLHDDLDDGDLEQ
AAV1          -----------------P--VV---RKRTYRKLCA---IHHLLGRAPE-IACSACDLVN----VDLDDCVSEQ
AAV2          -------------------------------------------------------------------------
AAV3          -----------------PVSVV---KKKTYQKLCP---IHHILGRAPE-IACSACDLAN----VDLDDCVSEQ
AAV4          -----------------PVSVV---RKRTYQKLCP---IHHIMGRAPE-VACSACELAN----VDLDDCDMEQ
AAV5          -----------------K---------------------------------ENLSD--F----GDFDDANKEQ
Brachylagus   ------------ES--SDQNES---DQKEYVKLCA---IYHLIV-----------VLVN----SPF-------
Lepus_am      ------------ES--SDQNES---DQKQYVKLCG---IYHLMV-----------VLVN----SPF-------
Oryctogalus   ------------ES--SDXNES---DQKEYVKLYVYYYIYYLIV-----------VLVN----SPF-------
S_audobonii   ------------ES--SDQNES---DQKEYVKLCA---IYHLIV-----------VLVN----SPF-------
S_floridanus  ------------ES--SDQNES---DQKEYVKLCA---IYHLIV-----------VLVN----SPF-------
bat           --------------------------------VPI---AEHDLCLAP----IEDSDFYV----GCIDDVNKEQ
bovineAAV     -----------------K---------------------------------ENVSD--F----NDFDDCNKEQ
caprine       -----------------K---------------------------------ENLSD--F----GDFDDANKEQ
duck          -----------------N----------------E---IEH-----------------V----FAIDDMENEQ
sealion       ----------------------------------------------------------V----SDPDDCTDEQ
simian        -----------------P--VV---RKKTYRKLCA---IHHLLGRAPE-IACSACDLVN----VDLDDCVSEQ
```

```
cons
```

B

```
AAAV          MR----------SYYEVIVQLPNDVESQVPGISDSFVNWITSREWTLPEDADWDLDQVDQVQLTLGDKIQREIRTHWGTMAKE-
AAV1          MP----------GFYEIVIKVPSDLDEHLPGISDSFVSWVAEKEWELPPDSDMDLNLIEQAPLTVAEKLQRDFLVQWRRVSKA-
AAV2          MP----------GFYEIVIKVPSDLDEHLPGISDSFVNWVAEKEWELPPDSDMDLNLIEQAPLTVAEKLQRDFLTEWRRVSKA-
AAV3          MP----------GFYEIVLKVPSDLDERLPGISNSFVNWVAEKEWDVPPDSDMDPNLIEQAPLTVAEKLQREFLVEWRRVSKA-
AAV4          MP----------GFYEIVLKVPSDLDEHLPGISDSFVSWVAEKEWELPPDSDMDLNLIEQAPLTVAEKLQREFLVEWRRVSKA-
AAV5          MA----------TFYEVIVRVPFDVEEHLPGISDSFVDWVTGQIWELPPESDLNLTLVEQPQLTVADRIRRVFLYEWNKFSKQ-
Brachilagus   MR----------TF-----AFPATGIEHLPGIPDTFVDWIATKNGLGGQRHGFKSKRATSA--DHHRKVHREFLKECRRLTKS-
Lepus_am      PR----------TF-----AFPATRTAHLPGIPDTFVDWIATKNGLXGQRHGFKSNRATSA--DHHRXVHREFLKECRRLTKS-
Oryctogalus   MR----------TF-----AFPATRTEHLPGIPDTFVDWMATKNGLXGQRHGFKSNRATSA--DHYREVHREFLKECRKLTKS-
S.floridanus  MR----------TF-----AFPATRTEHLPGIPDTFVDWIATKNGLRGQRHGFKSNRATSA--DHHRKVHRELLKECRRLTKS-
S_audobonii   MR----------TF-----AFPATRTEHLPGIPDTFVDWIATKNGLGGQRHGFKSNRAISA--DHHRKVHRDFLKECRRLTKS-
bat           ME-----------FYSIVLRLPGDFDSEVPGLQDSFYKWISGPRRELPEWSDLDPGQIESAYQILADKLVREFAQKWAAFSED-
bovineAAV     MA----------TFYEVIVRVPFDVEEHLPGISDNFVDWVTGQIWELPPESDLNLTLIEQPQLTVADRIRRVFLYEWNKFSKQ-
caprine       MA----------TFYEVIVRVPFDVEEHLPGISDSFVDWVTGQIWELPPESDLNLTLIEQPQLTVADRIRRVFLYEWNKFSKQ-
duck          MAFSRPLQISSDKFYEVIIRLPSDIDQDVPGLSLNFVEWLSTGVWE--PTGIWNMEHVNLPMVTLADKIKNIFIQRWNQFNQD-
sealion       MA----------SFYEVVAKIPSDLEEHLPGICDDFVSVIMSKEWRLPESSDLVLDQIDQPKLTIADAVRKVFYFEWLKYCRDV
simian        MP----------GFYEIVIKVPSDLDEHLPGISDSFVNWVAEKEWELPPDSDMDLNLIEQAPLTVAEKLQRDFLVKWRRVSKA-

cons                       :      .*      :**:   * . :                            : . :  .     .

AAAV          PDFHYFIQLEQGEVFFHLHVLLETCSVKPMVLGRYIRHIQQKIVSKVYCATSLRWKDGCVVTKTKN-FGGANKVRAESYIPAYL
AAV1          PEALFFVQFEKGESYFHLHILVETTGVKSMVLGRFLSQIRDKLVQTIYRGIEPTLPNWFAVTKTRNGAGGGNKVVDECYIPNYL
AAV2          PEALFFVQFEKGESYFHMHVLVETTGVKSMVLGRFLSQIREKLIQRIYRGIEPTLPNWFAVTKTRNGAGGGNKVVDECYIPNYL
AAV3          PEALFFVQFEKGETYFHLHVLIETIGVKSMVVGRYVSQIKEKLVTRIYRGVEPQLPNWFAVTKTRNGAGGGNKVVDDCYIPNYL
AAV4          PEALFFVQFEKGDSYFHLHILVETVGVKSMVVGRYVSQIKEKLVTRIYRGVEPQLPNWFAVTKTRNGAGGGNKVVDDCYIPNYL
AAV5          -ESKFFVQFEKGSEYFHLHTLVETSGISSMVLGRYVSQIRAQLVKVVFQGIEPQINDWVAITKVKK--GGANKVVDSGYIPAYL
Brachilagus   -NLNYFTQFEPSPCFYQNRRYEEQGFI-----AYHVPQIRNKLRISVAE-CRAQDSIGRPSSKIQI-TEPIRXEIRPIFLPT-L
Lepus_am      -NLNYLTQFEPSPRSYQNRRYEEQGSI-----ACHVPQMRNKLRISVSE-CRAQDSIGRPSSKIQI-KEPIRFKIRPMFLPT-L
Oryctogalus   -NLNNFTQFEPSPRFYQNRRYEEQGSI-----ACHVPQILNKFRISVSE-CRAQDSIGWPSSKIQI-KEPIRFEIRPIFLPT-L
S.floridanus  -NLNYFTQFEPSPCFYQNRRYEEQGFI-----AYHVPQIRNKLRISVAE-CRAQDSIGRPSSKIQIXTEPIRFEIRPIFLPT-L
S_audobonii   -NLNYFTQFEPSPCFYQNRRYEEQGFI-----ACHVPQIRNKLRISVAE-CRAQDSIGRPSSKIQI-KEPIRFEIRPIFLPT-L
```

```
bat          PRAPYFAQLEKGRENFHVHVLASSKKVGSFVVGRYVRKMRQHLVDVVFRKCEPVDADWLQVQKSGN--HKSNEIKDEGFIPAYL
bovineAAV    -ESKFFVQFEKGSEYFHLHTLVETSGISSMVLGRYVSQIRAQLVKVVFQNIEPRINDWVAITKVKK--GGANKVVDSGYIPAYL
caprine      -ESKFFVQFEKGSEYFHLHTLVETSGISSMVLGRYVSQIRAQLVKVVFQGIEPQINDWVAITKVKK--GGANKVVDSGYIPAYL
duck         -ETDFFFQLEEGSEYIHLHCCIAQGNVRSFVLGRYMSQIKDSILRDVYEGKQVKIPDWFSITKTKR--GGQNKTVTAAYILHYL
sealion      EEPLFFFQFEKGKENFHVHMVIETSNVSSMVLGRYIGTIKKKLVRKVFREVEPQMPDWLAVTKTKQ-SGGVNKTYDKGYIPAYL
simian       PEALFFVQFEKGESYFHLHILVETTGVKSMVLGRFLSQIRDKLVQTIYRGIEPTLPNWFAVTKTRNGAGGGNKVVDECYIPNYL

cons              : *:* .    : :       :     . .:  :   :   :               *        .      ::   *
```

End of frame “B”

```
AAAV         IPKQQPEVQWAWTNVPEYIKACLHRELRASLARLHFEEAGVSQS-KENLARTADGAPVMPTRVSKRYMELVDWLVEKGITTEKE
AAV1         LPKTQPELQWAWTNMEEYISACLNLAERKRLVAQHLTHVSQTQEQNKENLNPNSDAPVIRSKTSARYMELVGWLVDRGITSEKQ
AAV2         LPKTQPELQWAWTNMEQYLSACLNLTERKRLVAQHLTHVSQTQEQNKENQNPNSDAPVIRSKTSARYMELVGWLVDKGITSEKQ
AAV3         LPKTQPELQWAWTNMDQYLSACLNLAERKRLVAQHLTHVSQTQEQNKENQNPNSDAPVIRSKTSARYMELVGWLVDRGITSEKQ
AAV4         LPKTQPELQWAWTNMDQYISACLNLAERKRLVAQHLTHVSQTQEQNKENQNPNSDAPVIRSKTSARYMELVGWLVDRGITSEKQ
AAV5         LPKVQPELQWAWTNLDEYKLAALNLEERKRLVAQFLAESSQRSQ-EAASQREFSADPVIKSKTSQKYMALVNWLVEHGITSEKQ
Brachilagus  WVRANQNWQWAWTNMEAYIRAALPPVERERPVEDHPTDIPSKF---SPDSFSEDSGPVTRTQISQKYMAVVNWHVENGTTSGKP
Lepus_am     WESANQNWQWAWTNMEEYIRAALPPVERESPVEDRPTDVPSKF---SPDSFSEDSGPVTRTRISQKYMAVVNWHVENGTTSGKP
Oryctogalus  WERANQNWQWAWTNMEEYIRAALPLVERECPVEDRPTDVPSKF---SPDSFSEDSGPVTRTKISQKYMAVVNWHVENGTTSGKP
S.floridanus WVRANQNWQWAWTNMEAYIRAALPPVERERPVEDHPKDIPSKF---SPDSFSEDSGPVTRTQISQKYMAVVNWHVENGTTSGKP
S_audobonii  WERANQNWQWAWTNMEAYIRAALPPVERERPVEDHPTDIPSKF---PPDSFSEDSGPVTRTQISQKYMAVVNWHVENATTSGKP
bat          LPKRQSELQWAWTNIEKYERATLSVAERARLVEEWKRSLAAE----ESDPAEPERRPRKSTKSASEYMALVRWLVDNGIATERE
bovineAAV    LPKVQPELQWAWTNLEEYKLAALNLEERKRLVAQFQLESSQRSQ-EASSQRDVSADPVIKSKTSQKYMALVSWLVEHGITSEKQ
caprine      LPKVQPELQWAWTNLDEYKLAALNLEERKRLVAQFLAESSQRSQ-EAASQREFSADPVIKSKTSQKYMALVNWLVEHGITSEKQ
duck         IPKKQPELQWAFTNMPLFTAAALCLQKRQELLDAFQESEMNAV--VQEDQ-ASTAAPLISNRAAKNYSNLVDWLIEMGITSEKQ
sealion      LPKTQPELQWAWTNIEEYKSASLNLAERKRLVDEFLASLRRDGPSQSEPDDQQPHGPVIRNRTSQKYMALVSWLVENGITSEKQ
simian       LPKTQPELQWAWTNMEEYISACLNLAERKRLVAQHLTHVSQTQEQNKENLNPNSDAPVIRSKTSARYMELVGWLVDRGITSEKQ

cons             : : ***:**:  :  * *    *                            *   .: : .*  :* * :: . :: :
```
