# Supplementary material for: Evolution of dependoparvoviruses across geological timescales—implications for design of AAV-based gene therapy vectors
Source: Virus Evol. 2020 May 22;6(2):veaa043. doi: 10.1093/ve/veaa043 (PMC7474932; doi:10.1093/ve/veaa043)
Supplement: veaa043_Supplementary_Data [file ve_6_2_veaa043_s7.zip › FigS5.pdf]

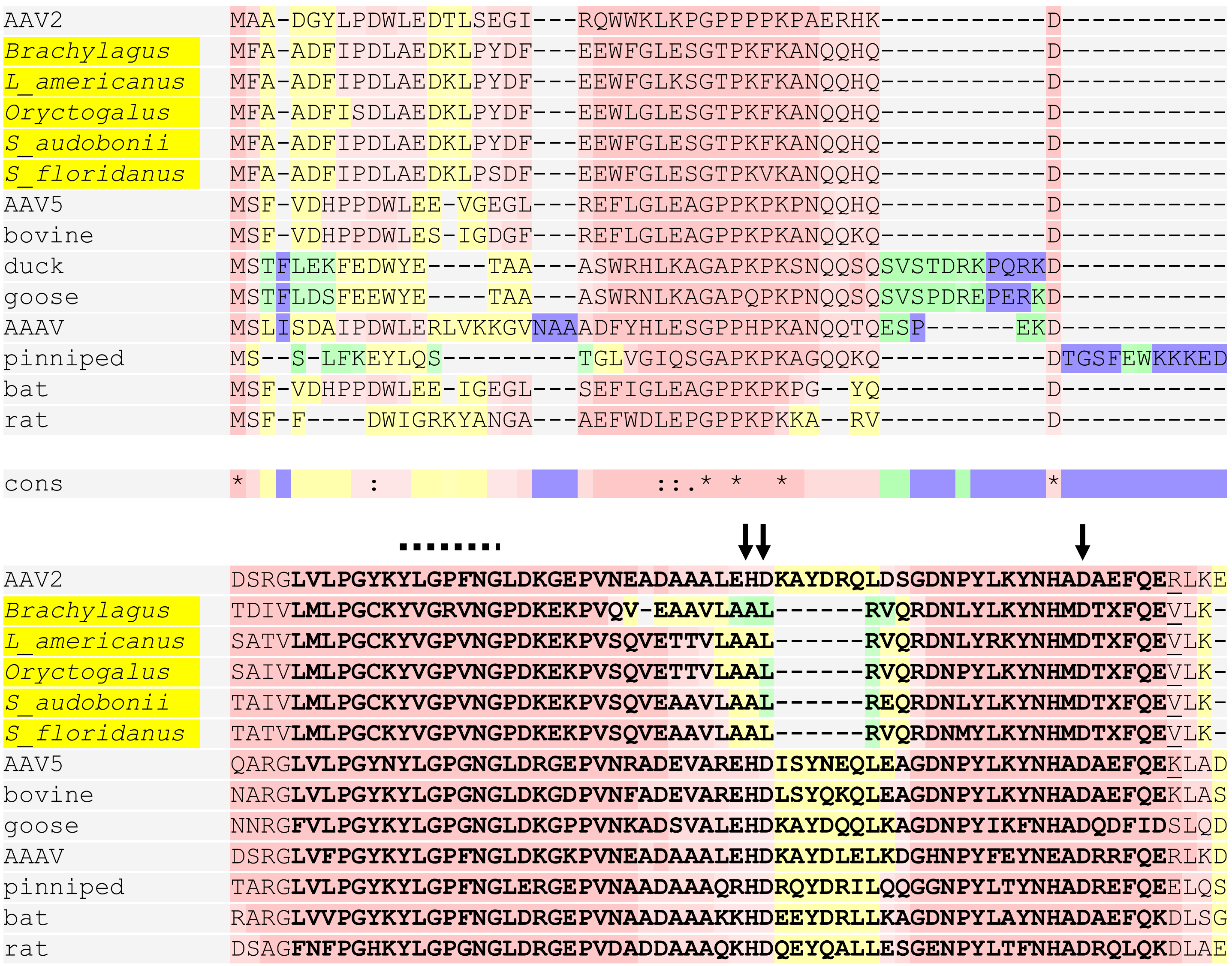

AAV2 MAA-DGYLPDWLEDTLSEGI---RQWWKLKPGPPPPKPAERHK-----------D-----------
Brachylagus MFA-ADFIPDLAEDKLPYDF---EEWFGLESGTPKFKANQQHQ-----------D-----------
L_americanus MFA-ADFIPDLAEDKLPYDF---EEWFGLKSGTPKFKANQQHQ-----------D-----------
Oryctogalus MFA-ADFISDLAEDKLPYDF---EEWLGLESGTPKFKANQQHQ-----------D-----------
S_audobonii MFA-ADFIPDLAEDKLPYDF---EEWFGLESGTPKFKANQQHQ-----------D-----------
S_floridanus MFA-ADFIPDLAEDKLPSDF---EEWFGLESGTPKVKANQQHQ-----------D-----------
AAV5 MSF-VDHPPDWLEE-VGEGL---REFLGLEAGPPKPKPNQQHQ-----------D-----------
bovine MSF-VDHPPDWLES-IGDGF---REFLGLEAGPPKPKANQQKQ-----------D-----------
duck MSTFLEKFEDWYE----TAA---ASWRHLKAGAPKPKSNQQSQSVSTDRKPQRKD-----------
goose MSTFLDSFEEWYE----TAA---ASWRNLKAGAPQPKPNQQSQSVSPDREPERKD-----------
AAAV MSLISDAIPDWLERLVKKGVNAAADFYHLESGPPHPKANQQTQESP------EKD-----------
pinniped MS--S-LFKEYLQS---------TGLVGIQSGAPKPKAGQQKQ-----------DTGSFEWKKKED
bat MSF-VDHPPDWLEE-IGEGL---SEFIGLEAGPPKPKPG--YQ-----------D-----------
rat MSF-F----DWIGRKYANGA---AEFWDLEPGPPKPKKA--RV-----------D-----------
cons * : ::.* * * *
AAV2 DSRGLVLPGYKYLGPFNGLDKGEPVNEADAAALEHDKAYDRQLDSGDNPYLKYNHADAEFQERLKE
Brachylagus TDIVLMLPGCKYVGRVNGPDKEKPVQV-EAAVLAAL------RVQRDNLYLKYNHMDTXFQEVLK-
L_americanus SATVLMLPGCKYVGPVNGPDKEKPVSQVETTVLAAL------RVQRDNLYRKYNHMDTXFQEVLK-
Oryctogalus SAIVLMLPGCKYVGPVNGPDKEKPVSQVETTVLAAL------RVQRDNPYLKYNHMDTXFQEVLK-
S_audobonii TAIVLMLPGCKYVGPVNGPDKEKPVSQVEAAVLAAL------REQRDNLYLKYNHMDTXFQEVLK-
S_floridanus TATVLMLPGCKYVGPVNGPDKEKPVSQVEAAVLAAL------RVQRDNMYLKYNHMDTXFQEVLK-
AAV5 QARGLVLPGYNYLGPGNGLDRGEPVNRADEVAREHDISYNEQLEAGDNPYLKYNHADAEFQEKLAD
bovine NARGLVLPGYKYLGPGNGLDKGDPVNFADEVAREHDLSYQKQLEAGDNPYLKYNHADAEFQEKLAS
goose NNRGFVLPGYKYLGPGNGLDKGPPVNKADSVALEHDKAYDQQLKAGDNPYIKFNHADQDFIDSLQD
AAAV DSRGLVFPGYKYLGPFNGLDKGKPVNEADAAALEHDKAYDLELKDGHNPYFEYNEADRRFQERLKD
pinniped TARGLVLPGYKYLGPFNGLERGEPVNAADAAAQRHDRQYDRILQQGGNPYLTYNHADREFQEELQS
bat RARGLVVPGYKYLGPFNGLDRGEPVNAADAAAKKHDEEYDRLLKAGDNPYLAYNHADAEFQKDLSG
rat DSAGFNFPGHKYLGPGNGLDRGEPVDADDAAAQKHDQEYQALLESGENPYLTFNHADRQLQKDLAE

```
cons              : .** :*:*  *. ::  **.  : ..               * *  ::. *  : . *

AAV2          DTSFGGNLGRAVFQAKKRVLEPLGLVEEPVK--TAP-GKKRPVEHSPV-EPDSSSGTGK-AGQQPA
Brachylagus   DTSSRGDLSKAVSQAKKWVVESFGLADEGN---MAL-GKKRPLPEE-K-TPEQAHGPGP-ESL---
L_americanus  DTSSRGDLSKAVSQAKKWVVESFGLVDEGN---MAL-GKKRPLPEE-K-TPEQAHGPGP-ESL---
Oryctogalus   DTSSRGDLSKAVSQAKKWIVESFGLVEESN---MAL-GKKRPLPEE-K-TLEQAHGSGP-ESL---
S_audobonii   DTSSRGDLSKAVSQAKKWVVESFGLADEGN---MAL-GKKRPLPEE-K-TPEQAHGPGP-ESL---
S_floridanus  DTSSRGDLSKAVSQAKKWVVESFGLADEGN---MAL-GKKRPLPEE-K-TPEQAHGPGP-ESL---
AAV5          DTSFGGNLGKAVFQAKKRVLEPFGLVEEGAK--TAP-TGKRIDDH------------------FPK
bovine        DTSFGGNLGKAVFQAKKRILEPLGLVETPDK--TAPAAKKRPLEQSPQ-EPDSSSGVGK-KGKQPA
goose         DQSFGGNLGKAVFQAKKRILEPFGLVEDPVN--TAP-AKKNTGKLTDH-YPVVKKPKLT-EEVSAG
AAAV          DTSFGGNLGKAIFQAKKRVLEPFGLIEQPDN--TAGTGEKRPERVDDF-FPKKKKAKTE-QGKAPA
pinniped      DESFGGNLGKAVFQAKKRILEPLGLVEEDPS--QP----------------DSSSD-----NTAPP
bat           DSSLAGNAANALFQAKKRVLEPFGLVEGEPEPKKTP-SVKRPH----A-SPDSSSGVGK-KGDQPA
rat           DQSFEGNLARGLFEAKKLAAQVVGV-EEPE---LAP-PVKRPHSP-EK-TPENQKGQPRPDPRTPA

cons          * *  *: ...: :***    : .*: :       .

AAV2          RKRLNFGQ------TGDADSVPDPQPL-GQ----PPAAPSGLGTNTMATGSGAPMADNNEGADGVG
Brachylagus   --KILFEKK-----KLNTVMLEQYPPH-TX----PPQMHLKLGSTTMSAGGGVPMGDNNHGAHGTQ
L_americanus  --KILFEKK-----KLNIVMLEQYPPH-TX----PPQMHLKLGSTTMSAGGGVPMGDNNQGAHGTQ
Oryctogalus   --KILFEKK-----KLNTVMLEQYPPH-TX----PPQMHLKLGSTTMSAGGGVPMGDNNQGTMGTQ
S_audobonii   --KILFEKK-----KLNTVMLEQYPPH-TX----PPQMHLKLGSTTMSAGGGVPMGDNNHGAHGTQ
S_floridanus  --KILFEKK-----KLNTVMLEQYPPH-TX----PPQMHLKLGSTTMSAGGGVPMGDNNHGAHGTQ
AAV5          RKKARTEEDSKPSTSSDAEAGPSGSQQLQI----PAQPASSLGADTMSAGGGGPLGDNNQGADGVG
bovine        RKRLNFDD------EPG---AGDGPPP-EG-----PSSGAMSTETEMRAAAGGNGGDAGQGAEGVG
goose         GG------------------SSAVQDG-GA----TAEGTEPVAASEMAEGGGGAMGDSSGGADGVG
AAAV          QTGED---------------PGEGTSS-NA----GSSAPSSVGSSVMAEGGGGPMGDAGQGADGVG
pinniped      VKKSRLEE-------AQ---PIQSP-D-VSSSTGGGIADVMSGDAEMAAVGGGAPGVDGQGAEGVG
bat           RKRLDFGT------EPA-----GQDGA-GR----AAQAAGDMASAEVAAGGGGPVGDDAQGADGVG
rat           KKRLEFSDQ--P---GSSADLPASSQQ-SQ----PPAGVPGVVPGTMSAGGGAPVDDAQQGADGVG
```

```
cons                                                        :   ..        *: *.
                                                       VRI
AAV2          NSSGNWHCDSTWMGDRVITTSTRTWALPTYNNHLYKQISSQSG--ASNDNHYFGYSTPWGYFDFNR
Brachylagus   MGT-------EVSNGWRTTMSTRTWVLSSYNNYLYQQISSIRT--ARNVSAHFRCSTSWGYL--NR
L_americanus  MGT-------EVPNGW-RTVSTRTWVLSSYNNYLYQQISSIRT--ARNVSAHFRCSTSWGYL--NK
Oryctogalus   MGT-------EVPNGWGTTMSTRTWVLSSYNNYLYQQISSIRT--ARNVSAHFRCSTSWEYL--NR
S_audobonii   MGT-------EVSNGWRTTMSTRTWVLSSYNNYLYQQISSIRT--ARNVSAHFMCSTSWGYL--NR
S_floridanus  MGT-------EVSNGWRTTMSTXTWVLSSYNNYLYQQISSIRT--ARNVSAHFRCSTSWGYL--NR
AAV5          NASGDWHCDSTWMGDRVVTKSTRTWVLPSYNNHQYREIKSGSVD-GSNANAYFGYSTPWGYFDFNR
bovine        NASGDWHCDSTWSESHVTTTSTRTWVLPTYNNHLYLRLGSSN-----ASDTFNGFSTPWGYFDFNR
goose         NASGNWHCDSQWMGNTVITKTTRTWVLPSYNNHIYKAITSGTS--QDANVQYAGYSTPWGYFDFNR
AAAV          NSSGNWHCDSQWLDNGVVTRTTRTWVLPSYNNHLYKRIQGPGG--TDPNNKFFGFSTPWGYFDYNR
pinniped      TSSGNWHCDSQWSEGHVRTTSTRTWVLPSYNNHLYKRLGSSA-----QSNTYNGFSTPWGYLDFNR
bat           NASGNWHYDSVWMDGAVITKSTRTWSLPAYNNHLYRQIQSSGT----GDGTYFGYSTPWGYFDFNR
rat           NASGDWHCDSKWLGNRVLTRSTRTWVLPSYNNHLYKQISDASGVHSLPGSRYFGYSTPWGYFDFNR

cons           .:           .   *  :* ** *.:***: *  :             .   **.* *:  *:

                                                     VRII
AAV2          FHCHFSPRDWQRLINNNWGFRPKRLNFKLFNIQVKEVTQNDGTTTIANNLTSTVQVFTDSEYQLPY
Brachylagus   YHYHFSLXDWQRLINNHXGFCPKTL--RVFXIQVKDV--------LANNLTPMVHIFADSEYQVPL
L_americanus  FHCHCSLRDWQSLINNHWGICPKTLKVRVFNIQVKEVM------TLANNLTPMVH--ADSEYQVPY
Oryctogalus   FHCHCSLRDWQRLINNHWGVCPKTLKVRVFNIQVKEVM------TLANNLTPMVHVFADSEYQVPY
S_audobonii   YHCHFSLXDWQRLINNHWGFCPKTL--RVFNIQVKDVM------TLANNLTPMGHVFADSEYQVLY
S_floridanus  YHCHFSLXDWQRLINNHWGFCPKTL--RVFNIQVKDVM------TLANNLTPMVHVFADSEYQVLY
AAV5          FHSHWSPRDWQRLINNYWGFRPRSLRVKIFNIQVKEVTVQDSTTTIANNLTSTVQVFTDDDYQLPY
bovine        FHCHFSPRDWQRLINNHWGLRPKSMQVRIFNIQVKEVTTSNGETTVSNNLTSTVQIFADSTYELPY
goose         FHCHFSPRDWQRLINNHWGIRPKSLKFKIFNVQVKEVTTQDQTKTIANNLTSTIQVFTDDEHQLPY
AAAV          FHCHFSPRDWQRLINNNWGIRPKAMRFRLFNIQVKEVTVQDSNTTIANNLTSTVQVFADKDYQLPY
pinniped      WHCHFSPRNWQRLINNNWGIRPKRLNVKLFNIQVKEVTTEGGTTTVANNLTSTIQVFADNAYELPY
bat           FHCHFSPRDWQRLINNHWGIRPKRLHFKLFNIQVKEVTTTDGTTTIANNLTSTIQVFADTEYQLPY
rat           FHCHFSPRDWQRLVNNHWGFRPKRLRVKLFNIQVKEVTTTDSTTTVSNNLTSTVQVFTDDEYQLPY
```

```
cons           :* * *  :** *:**  *. *: :  ::* :**: *        ::****.  :  :*  :::  

                                          VRIII
AAV2           VLGSAHQGCLPPFPADVFMVPQYGYLTLN---NG-------SQAVGRSSFYCLEYFPSQMLRTGNN
Brachylagus    PSGQCNRGCLLVFLQ----RPLHSCSDDD---NT-------DSPIDRSTLYSLVYFPSKMSKPSNN
L_americanus   LLSSAIGGCLPVFLQ----RPLHSCNDDD---NT-------DSPIDRSTLYSLVYFPSKMSKPSNN
Oryctogalus    LLGSATGGCLPVFLQ----RPLHSCSDDD---NT-------DSPIDRSTLYSLVYFPGKMLKPSNN
S_audobonii    LLGSANRGCLLVFLQ----RPLHSCSDDD---NT-------DSPIDRSTLYSLVYFPSKMSKPSNN
S_floridanus   LLGSANRGCLLVFLQ----RPLHSCSDDD---NT-------DSPIDRSTLYSLVYFPSKMSKPSNN
AAV5           VVGNGTEGCLPAFPPQVFTLPQYGYATLNRD-NT-------ENPTERSSFFCLEYFPSKMLRTGNN
bovine         VMDAGQEGSLPPFPNDVFMVPQYGYCGLVTGGSS-------QNQTDRNAFYCLEYFPSQMLRTGNN
goose          VLGSATEGTMPPFPSDVYALPQYGYCTMHTNQNG-------ARFNDRSAFYCLEYFPSQMLRTGNN
AAAV           VLGSATEGTFPPFPADIYTIPQYGYCTLNY---N-------NEAVDRSAFYCLDYFPSDMLRTGNN
pinniped       VVDAGHEGALPPFPNDVFMIPQYGYCGLVSG-QS-------QNQSDLCSFYCLEYFPSQMLRTGNN
bat            VLGNAHEGCLPPFPADVFMLPQYAYLTLN---ANPTNQGAAALSLPQSAFYCLEYFPSQMLRTGNN
rat            VCGNATEGCLPPFPPDVFTLPQYGYATLNDS-NN-------GNPTNRSSFFCLEYFPSKMLRTGNN

cons             .    * :  *       * :.                        :::.* ***..* :..*.

                                                          VRIV
AAV2           FTFSYTFEDVPFHSSYAHSQSLDRLMNPLIDQYLYYLSRTNTPS-GTT-TQSRLQFSQAGASDIRD
Brachylagus    SEFTFKFEEVALYSRFVPSELFNKLAILQVDQYLWKLSIQEHPS-S-------KSF------QLGK
L_americanus   SEFTFQFEEVALYSRFVPSELFNKLANLQVDQYLWKLSIQEHPS-S-------KSF------QLGK
Oryctogalus    SEFAFQFEEVALYSRFVPSELFNKLANLQVDQYLWKLSIQEHPS-S-------KSF------QLGK
S_audobonii    SEFTFKFEEVAFYSRFVPSELFXKLANLQVDQYLWKLSIQEHPS-S-------KSF------QLGK
S_floridanus   SEFTFRFEEVALYSRFVPSELFNKLANLQVDQYLWKLSIQEHPS-S-------KSF------QLGK
AAV5           FEFTYNFEEVPFHSSFAPSQNLFKLANPLVDQYLYRFVSTNNTG-G-------VQFNKNLAGRYAN
bovine         FEMVYKFENVPFHSMYAHSQSLDRLMNPLLDQYLWELQSTTSGG-TLNQGNSATNFAKLTKTNFSG
goose          FEFTFDFEEVPFHSMFAHSQDLDRLMNPLVDQYLWNFNEVDSSR--------NAQFKKAVKGAYGT
AAAV           FEFTYTFEDVPFHSMFAHNQTLDRLMNPLVDQYLWAFSSVSQTG-SSG---RALNYSRATKTNMAT
pinniped       FEMNFRFEDVPFHSMYAHSQSLDRLMNPLIDQYLWSLKQTGNPT-GST--TRDLKFIKNKVPNFAH
bat            FSFSYEFEKLPFHSMFMHSQSLDRLMNPLIDQYLWYLNATTGN---------NLSFNKAGAKNFPE
rat            FEFTFEFEDVPFHTGFAPCQNLFKLSNPLVDQYLYRFQGTDNSS-SST--PGVVKFEKCVAHNYAQ
```

```
cons         : : **.:.::  :   : : :*   :****: :                     :

                                    VRV                            VRVI
AAV2         QSRNWLPGPCYRQQRVSKTSA--D--NNNSE-----YSWTGATKYHLNGRDSLVNPGPAMA-SHKD
Brachylagus  TYQNRLSGAVYKTQAQNTAXE--QI-STKLS----AATTDSS-----NNYNYTLKTRTRYN-ITHQ
L_americanus TYQNRLSGAVYKTQAQNTAXE--QI-STKLS----AATIDSS-----NNYSY-LKTKTRYN-VTHQ
Oryctogalus  TYQNSLSRAVYKTQAQNTAXE--QI-STKLS----AATIDSS-----NNYNYTLKTRTRYIIVTHQ
S_audobonii  TYQNRLSGTVYKTQAQNTAXE--QI-STKLS----AATIDSS-----NNYNYTLKTRTRYN-ITHQ
S_floridanus TYQNRLSGAVYKTQAQNTAXE--QI-STKLS----AATTDSS-----NNYNYTLKTRTRYN-ITHQ
AAV5         TYKNWFPGPMGRTQGWNLGSG--VN--RASV-----SAFATTNRMELEGASYQVPPQPNGM-TNNL
bovine       YRKNWLPGPMMKQQRFSKTAS--Q--NYKIPQGRNNSLLHYETRTTLDGRWSNFAPGTAMA-TAAN
goose        MGRNWLPGPKFLDQRVRAYTGGTD--NYANW-----NIWSNGNKVNLKDRQYLLQPGPVSA-TYTE
AAAV         QYRNWLPGPFVRDQQIFTGASNIT--QNNVF-----NVWDKGKQWVIDNRINMMQPGPAAA-TTFS
pinniped     YGKNWLPGPFIRQQGWTTQNI--N--NSVV---NFNDMLGKNSTFTLDTRWSSLAPGPCMG-DDGR
bat          YFRNWLPGPGQRVQQWSTIGT--Q--NNAQT-----GTWASANKWILMGRSSKMAPGLAQP-V--R
rat          QYKNWLTGAFQRNQDYNYVSG--TSN-YKGV-----VGSNQNNLQRIENVQFAIAPDVPSM-CNHL

cons          :*  :.  .          *                                         .

                        VRVII
AAV2         D--EEKFFPQSGVLIFGKQGS--EK-TN---VDIEKVMITDEEEIRTTNPVATEQYGSVSTNLQRG
Brachylagus  D--GTNFVPRTSPXFVNDDKTALRD-TS--FLLFLSYLLLDDSETTATSPQAYNNADRVTTDNQSV
L_americanus D--GTNFVPRTSPXFFNDDKTALGD-TP--LLLFLSYLLLDESETTATSPQAYNA-DRVTTNNQSV
Oryctogalus  D--GTNFVPRTSPXFFNDDKIVLRD-TP--LLLFLSYLLLDESVTTATSPQVYNNADRVTTNNQSV
S_audobonii  D--GTNFVPRTSPXFVNDDKTALRD-TS--LLLFLSYLLLDDSETTATSPQAYSNADRVTTDNQSV
S_floridanus D--GTNFVPRTSPXFVNDDKTALRD-TS--FLLFLSYLLLDDSETTATSPQAYNNADRVTTDNQSV
AAV5         QG--SNTYALENTMIFNSQPANPGT-TA--TYLEGNMLITSESETQPVNRVAYNVGGQMATNNQSS
bovine       D--ATDF-S-QAQLIFAGPNIT-GN-TT---TDANNLMFTSEDELRATNPRDTDLFGHLATNQQNA
goose        G--EASSLPAQNILGIAKDPYRSGS-TT---AGISDIMVTEEQEVAPTNGVGWKPYGRTVTNEQNT
AAAV         G--EPDRQAMQNTLAFSRTVYDQTT-ST---TDRNQLLITNEDEIRPTNSVGIDTWGVVPNNNQSK
pinniped     TPSTTKF-S-NAQLMFGSGT-Q--P-TE---GGEDAVHITSESEVKATNPTAIDEYGRVADNTQNA
bat          N--A-QTVTNGSQLIFNNETI--KGSTATASTVHSGLLVTNESETAPTNPNSATKWGVMTDNQQTT
rat          EG--TNMIALDNSLIFRDVSTAPGD-TT--QYNINQVIVTSEAETQSVNAYSGDTCGRIVNNSQNS
```

```
cons                       .                      .  :    ...       .    . *

                             VRVIII
AAV2              NRQAATADVNTQGVLPGMVWQDRDVYLQGPIWAKIPHTDGHFHPSPLMGGFGLKH-PPP-QILIKN
Brachylagus       STALRTGLFNHXGTIAGSAXMSRDIYHQGPIXTKIPITGEHFHPSPHLGEFGLKI-PPP-MLLIKN
L_americanus      INSSXNRSFNQXGTKAGSAQMSRDIYHQGPIXTKIPNTGEHFHPSPHLGEFGLKK-PPP-MLLIKN
Oryctogalus       STALRTGLFNHXGTIPGPAWMSXDIYHQGPIXTKIPIPKEHFHPSPHLGEFGLKK-KPPRMLLIKN
S_audobonii       STALRTGLFNHXGTIAGSVXMSRRIYHQGPIXTKIPIPGEHFHPSPHLGEFGLKNIPPP-MLLIKN
S_floridanus      STALRTGLFNHXGTIAGSAXMSRDIYHQGPIXTKIPIPGEHFHPSPHLGEFGLKNIPPP-MLLIKN
AAV5              TTAPATGTYNLQEIVPGSVWMERDVYLQGPIWAKIPETGAHFHPSPAMGGFGLKH-PPP-MMLIKN
bovine            TTVPTVDDVDGVGVYPGMVWQDRDIYYQGPIWAKIPHTDGHFHPSPLIGGFGLKS-PPP-QIFIKN
goose             TTAPTSSDLDVLGALPGMVWQNRDIYLQGPIGAKIPKTDGKFHPSPNLGGFGLHN-PPP-QVFIKN
AAAV              VTAGTRAAINNQGALPGMVWQNRDIYLQGPIWAKIPDTDNHFHPSPLIGGFGCKH-PPP-QIFIKN
pinniped          TTAPTTVGNAAMGAMPGMVWQDRDIYLQGPIWGKIPHTDGHFHPSPLMGGFGYRK-PPP-QIFIKN
bat               STTPTVSDDLEAHVFPGMVWQDRDIYLQGPIWAKIPETDGHFHPSPLMGGFGLKN-PPP-QILVKN
rat               GTNAGTTGINYKGTMPSSVWMDRDVYLQGPIWAKIPHTGAHFHPSPMMGGFGLRN-PPP-MMLIKN

cons                             .. .  .  :* ** *  *** .  : **** :* ** :   **  :::**

                             VRVIII
AAV2              TPVPANPSTTFSAAKFASFITQYSTGQVSVEIEWELQKENSKRWNPEIQYTSNYNK--SVNVDFTV
Brachylagus       V--PGNV-TAFTQ-MGNLLISQYATISVPMEMTXRLREENSKRXNPEMQDTNNYKS--PTYVDFVP
L_americanus      MPVPGNV-TAFTE-KGNLLITQYATISVPMEMTXRLREENSKRXNPEMQDTNNYKS--PTXVDFVP
Oryctogalus       V--PGNV-TAFTQ-IGNLLITQYATISVPMEMTXRLREEISKRXNPEMQDTNNYKS--PTYVDFVP
S_audobonii       V--PGNV-TAFTQ-MGNLLISQYATISVPMEMTLRLREENSKRXNPEMQDTNNYKS--PTYVDFVP
S_floridanus      V--PGNV-TAFTQ-MGNLLISQYATISVPMEMTXRLREENSKRXNPEMQXTNNYKS--PTYVDFVP
AAV5              TPVPGNI-TSFSDVPVSSFITQYSTGQVTVEMEWELKKENSKRWNPEIQYTNNYND--PQFVDFAP
bovine            TPVPANPATTFSPARINSFITQYSTGQVAVKIEWEIQKERSKRWNPEVQFTSNYGA--QDSLLWAP
goose             TPVPADPPVEYVHQKWNSYITQYSTGQCTVEMVWELRKENSKRWNPEIQFTSNFSN--RTSIMFAP
AAAV              TPVPANPSETFQTAKVASFINQYSTGQCTVEIFWELKKETSKRWNPEIQFTSNFGN--AADIQFAV
pinniped          TPVPGNPATTFSPNRINNFITQYSTGQVTVTIDWELQKENSKRWNPEVQFTSNFGT--VDSLNWAP
bat               TPVPAVPPTTFTPQKVNSFITQYSTGQVTVEIEWELRKEKSKRWNPEIQYTSNFEN--SANVQFSV
rat               TPVPGNV-TTFTEVKVNQFITQYSTGQITVDVEWELQKENSKRWNPEIQYTNNYSN--NTFVDFAP
```

```
cons           *.     :        *.**:* . .: :  .:::* *** ***:* :.:          :


AAV2           DTNGVYSEPRPIGTRYLTRNL
Brachylagus    SDQGVYRSTRTT---------
L_americanus   SDQGVYRSTRTT---------
Oryctogalus    SDQGVYRSTRTT---------
S_audobonii    SDQGVYRSTRTT---------
S_floridanus   SDQGVYRSTRTT---------
AAV5           DSTGEYRTTRPIGTRYLTRPL
bovine         DNAGAYKEPRAIGSRYLTNHL
goose          NETGGYVEDRLIGTRYLTQNL
AAAV           SDTGSYSEPRPIGTRYLTKPL
pinniped       DNAGNYKEPRVVGSRFLTHIL
bat            NGDGAYIEPRPIGTRYLTHN-
rat            NANGDYQMTRPIGTRYPTRPP

cons           .  * *   *
```
