# Supplementary material for: Evolution of dependoparvoviruses across geological timescales—implications for design of AAV-based gene therapy vectors
Source: Virus Evol. 2020 May 22;6(2):veaa043. doi: 10.1093/ve/veaa043 (PMC7474932; doi:10.1093/ve/veaa043)
Supplement: veaa043_Supplementary_Data [file ve_6_2_veaa043_s7.zip › S1 Table.docx]

S1 Table- Whippomorpha Species and Specimen Source Id

| Whippomorpha |  | |
| --- | --- | --- |
| Species | | Individual Specimen Sequence Source |
| *Balaena mysticetus* | | UAM:Mamm:86892 |
|  | | UAM:Mamm: 86893 |
|  | | UAM:Mamm:97497 |
|  | | UAM:Mamm:99854 |
| *Balaenoptera acutorostrata* | | UAM:Mamm:85669 |
| *Balaenoptera physalus* | | Cell Line Lab # 17518 Sand Diego Global |
| *Delphinapterus leucas* | | UAM:Mamm:36490 |
|  | | UAM:Mamm:91765 |
|  | | UAM:Mamm:115628 |
|  | | UAM:Mamm:30668 |
| *Delphinus capensis* | | Stranding Network, Mystic Aquarium |
| *Delphinus delphis* | | UAM:Mamm:90366 |
|  | | UAM:Mamm:90370 |
| *Eschrichtius robustus* | | UAM:Mamm:90177 |
|  | | UAM:Mamm:117578 |
| *Lagenorhynchus obliquidens* | | UAM:Mamm:85640 |
|  | | UAM:Mamm:90232 |
|  | | UAM:Mamm:90373 |
| *Lipotes vexillifer* | | NCBI assembly  NW_006780611.1 |
| *Lissodelphis borealis* | | UAM:Mamm:90251 |
|  | | UAM:Mamm:90324 |
| *Megaptera novaeangliae* | | UAM:Mamm:30661 |
|  | | UAM:Mamm:87413 |
|  | | UAM:Mamm:120796 |
| *Mesoplodon stejnegeri* | | UAM:Mamm:85500 |
|  | | UAM:Mamm:118781 |
|  | | UAM:Mamm:118820 |
| *Orcinus orca* | | UAM:Mamm:34219 |
|  | | UAM:Mamm:85635 |
|  | | UAM:Mamm:113864 |
| *Phocoena phocoena* | | UAM:Mamm:70329 |
|  | | UAM:Mamm:118788 |
|  | | UAM:Mamm:36428 |
| *Phocoenoides dalli* | | UAM:Mamm:90220 |
|  | | UAM:Mamm:97800 |
|  | | UAM:Mamm:90318 |
| *Physeter macrocephalus* | | Physeter catodon NW_006712748.1 |
| *Stenella coeruleoalba* | | UAM:Mamm:90272 |
|  | | UAM:Mamm:90273 |
|  | | UAM:Mamm:90358 |
| *Tursiops truncatus* | | NIST Tur_tru v1 Assembly GCF_001922835.1 |
|  | | Cell Line Lab # 12580-Sand Diego Global |
| *Ziphius cavirostris* | | UAM:Mamm:90300 |
| *Hippopotamus amphibius* | | ASM299558v1 Assembly GCA_002995585.1 |
