# Supplementary material for: Evolution of dependoparvoviruses across geological timescales—implications for design of AAV-based gene therapy vectors
Source: Virus Evol. 2020 May 22;6(2):veaa043. doi: 10.1093/ve/veaa043 (PMC7474932; doi:10.1093/ve/veaa043)

S2 Fig. RNA-seq reads mapping to EVE region with AAV2 annotation overlay for (A) Fin whales and (B) Domestic rabbit.


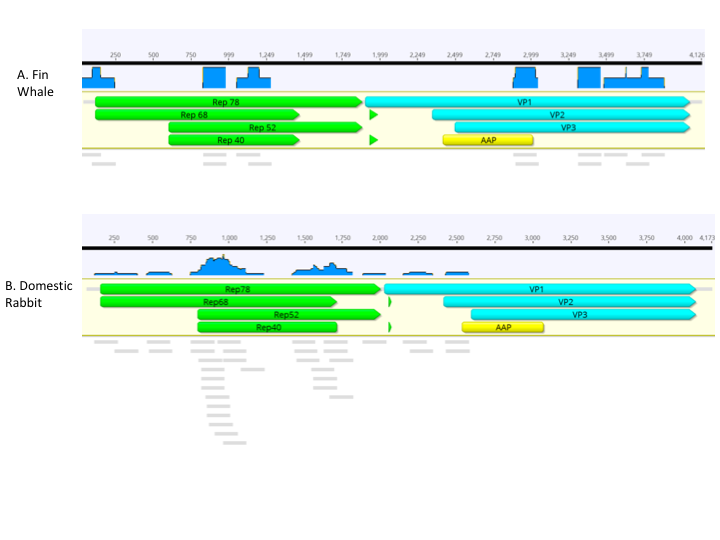

Supplement: veaa043_Supplementary_Data [file ve_6_2_veaa043_s7.zip › S2 Fig.docx]
