# Supplementary material for: Evolution of dependoparvoviruses across geological timescales—implications for design of AAV-based gene therapy vectors
Source: Virus Evol. 2020 May 22;6(2):veaa043. doi: 10.1093/ve/veaa043 (PMC7474932; doi:10.1093/ve/veaa043)
Supplement: veaa043_Supplementary_Data [file ve_6_2_veaa043_s7.zip › S2 Table.docx]

| S2 Table- Chiroptera Species and Specimen Source Id for EVE-positive and EVE-negative samples  Chiroptera | |
| --- | --- |
| Species | Individual Specimen Sequence Source |
| KIF-EVE Positive Bats |  |
| *Eptesicus andinus* | MSB:Mamm:141340 |
| *Eptesicus fuscus* | EptFus1.0 Assembly GCA_000308155.1 |
| *Lasionycteris noctivagans* | MSB:Mamm:156703 |
| *Myotis brandtii* | NCBI ASM41265v1 Assembly GCA_000412655.1 |
| *Myotis davidii* | NCBI Assembly ASM32734v1 GCA_000327345.1 |
| *Myotis lucifugus* | Myoluc2.0 Assembly GCA_000147115.1 |
| *Myotis velifer incautus* | Cell Line ATCC; catalogue no. CRL-6012 |
| *Plecotus ognevi* | MSB:Mamm:267244 |
|  |  |
| Kif-EVE Negative Bats |  |
| *Eidolon helvum* | GCA_000465285.1 ASM46528v1 |
| *Hipposideros armiger* | GCA_001890085.1 ASM189008v1 |
| *Megaderma lyra* | GCA_000465345.1 ASM46534v1 |
| *Miniopterus natalensis* | Mnat.v1 Assembly  GCA_001595765.1 |
| *Natalus stramineus* | MSB:Mamm:212701 |
| *Noctilio leporinus* | MSB:Mamm:268423 |
| *Nyctinomops macrotis* | MSB:Mamm:124700 |
| *Pteropus alecto* | GCA_000325575.1 ASM32557v1 |
| *Pteronotus parnellii* | GCA_000465405.1 ASM46540v1 |
| *Pteropus vampyrus* | GCA_000151845.2 Pvam_2.0 |
| *Rhinolophus ferrumequinum* | GCA_000465495.1 ASM46549v1 |
| *Rhinolophus sinicus* | GCA_001888835.1 ASM188883v1 |
| *Rousettus aegyptiacus* | GCA_001466805.2 Raegyp2.0 |
| *Rousettus amplexicaudatus* | MSB:Mamm:93248 |
| *Saccopteryx bilineata* | MSB:Mamm:155915 |
| *Tadarida brasiliensis* | MSB:Mamm:278417 |
