# Supplementary material for: Evolution of dependoparvoviruses across geological timescales—implications for design of AAV-based gene therapy vectors
Source: Virus Evol. 2020 May 22;6(2):veaa043. doi: 10.1093/ve/veaa043 (PMC7474932; doi:10.1093/ve/veaa043)
Supplement: veaa043_Supplementary_Data [file ve_6_2_veaa043_s7.zip › S3 Table.docx]

S3 Table- Lagomorpha Species and Specimen Source Id

| Lagomorpha | |
| --- | --- |
| Species | Individual Specimen Sequence Source |
| Brachylagus idahoensis | MSB:Mamm:281514 |
|  | MSB:Mamm:281525 |
| Lepus americanus | MSB:Mamm:214797 |
|  | MSB:Mamm:285149 |
|  | MSB:Mamm:224796 |
| Lepus californicus | MSB:Mamm:278445 |
|  | MSB:Mamm:28106 |
| Lepus europeaus | MSB:Mamm:236931 |
| Ochotona princeps | NCBI OchPri3.0 Assembly GCA_000292845.1 |
| Oryctolagus cuniculus | MSB:Mamm:265752 |
|  | Cell Line RK13 ATCC® CCL-37 |
|  | OryCun2.0 Assembly  GCA_000003625.1 |
| Sylvilagus audubonii | MSB:Mamm:265624 |
|  | MSB:Mamm:278405 |
| Sylvilagus floridanus | MSB:Mamm:85845 |
|  | MSB:Mamm:92747 |
|  | Cell Line SF1 ATCC® CCL-68 |
