# Supplementary material for: Evolution of dependoparvoviruses across geological timescales—implications for design of AAV-based gene therapy vectors
Source: Virus Evol. 2020 May 22;6(2):veaa043. doi: 10.1093/ve/veaa043 (PMC7474932; doi:10.1093/ve/veaa043)
Supplement: veaa043_Supplementary_Data [file ve_6_2_veaa043_s7.zip › S4 Table.docx]

S4 Table-Summary of the indels for the variable regions (VRs) and the phospholipase A2 (PLA2) domain in representative endogenous dependoparvoviral elements of each mammalian taxa studied, compared to those of the type virus of genus *Dependoparvovirus*, adeno-associated virus 2.

|  | PLA2 | VR1 | VR2 | VR3 | VR4 | VR5 | VR6 | VR7 | VR8 | VR9 |  |
| --- | --- | --- | --- | --- | --- | --- | --- | --- | --- | --- | --- |
| *Hippopotamus* | +33 | -2 | N/A | +4 | -6 | N/A | N/A | -6 | N/A | Absent^1^ | Whippomorha |
| *Balaneoptera* | +1 | -3 |  |  |  |  |  |  |  | N/A |  |
| *Physeter* |  | -2 |  |  |  |  | +8 |  |  |  |  |
| *Mesoplodon* |  |  |  |  |  |  | Masked by CHR-2A SINE insertion |  | +4 |  |  |
| *Ziphius* |  |  |  |  |  |  |  |  | N/A |  |  |
| *Lipotes* | +30 |  |  |  |  |  |  |  |  |  |  |
| *Turciops* | +1 |  |  |  |  |  |  |  |  |  |  |
| *Orca* |  |  |  |  |  |  |  |  |  |  |  |
| *Lepus* | -6^2^ | N/A | -6^3^ | -4 | -13 | +2 | N/A | +3 | N/A | -2 | Leporidae |
| *Sylvilagus* |  |  |  |  |  |  |  |  |  | -4 |  |
| *Brachylagus* | -7^2^ |  | -8^3^ |  |  |  |  |  |  |  |  |
| *Oryctogalus* | -6^2^ |  | -6^3^ |  |  |  | +1 |  |  |  |  |
| *Eptesicus fuscus* | N/A | +1 | N/A | -1 | +1 | -1 | -1 | N/A | N/A | N/A | Vespertilionidae |
| *E. andinus* | -5 |  |  |  | Absent | Absent |  |  |  |  |  |
| *Lasionycteris* | N/A |  |  |  | -4 | -1 |  |  |  |  |  |
| *Myotis brandtii* |  |  | -2 |  | -4 |  |  |  |  |  |  |
| *M. davidii* |  |  |  |  | -5 |  |  |  |  |  |  |
| *Plecotus* |  |  | N/A |  | -6 | N/A |  |  |  |  |  |

^1^Absent along with the entire C-term coding sequence

^2^The entire catalytic domain is absent

^3^The entire VR2 is deleted
