# Supplementary material for: Evolution of dependoparvoviruses across geological timescales—implications for design of AAV-based gene therapy vectors
Source: Virus Evol. 2020 May 22;6(2):veaa043. doi: 10.1093/ve/veaa043 (PMC7474932; doi:10.1093/ve/veaa043)
Supplement: veaa043_Supplementary_Data [file ve_6_2_veaa043_s7.zip › S5 Table.docx]

| S5 Table- Primers for amplification of targeted loci for sequencing and generation of phylogenetic trees | | | |
| --- | --- | --- | --- |
|  | Gene | Primer Name | Sequence 5’ to 3’ |
| Cetacean |  |  |  |
|  | Mitochondrial | 12sc | AAGGCTGGGACCAAACCTT |
|  |  | L15812 | CCTCCCTAAGACTCAAGGAAG |
|  | Actin | ACT3-F | GGTTATCTGATGTATTCC |
|  |  | ACT1385-R | CTTGTGAACTGATTACAGTCC |
|  |  | Cet Actin intron-r | AAACAGCCTTAGTTGATTG |
|  |  | Cet Actin int intron-f | AGGTCACTCACTCTCACAG |
|  | vWF | vWF-A | CTGTGATGGTGTCAACCTCACCTGTGAAGCCTG |
|  |  | vWF-B | TCGGGGGAGCGTCTCAAAGTCCTGGATGA |
|  |  | vWF-B2 | GCAGGGTTTCCTGTGACCATGTAGACCAG |
|  | Cetacean EVE | Cet Nest Int VP1 f | GGTTCAGGAATTACACGGTTTATAGAGGGATC |
|  |  | Cet again out vp1 r | CATGGCAATGCTATACCGGT |
|  |  | Cet Flank EVE (out) f | AGCTCTGTCACGGCTAAACT |
|  |  | Cet Flank EVE (out) r | TGTATACACCTGTGTAGCG |
| Leporidae |  |  |  |
|  | Mitochondrial | LI4724B | CGAGATCTGAAAAACCATCGTTG |
|  |  | H15915a | AACTGCAGTCATCTCCGGTTTACAAGAC |
|  |  | RabCytb F | ATGACCAACATYCGTAAAAC |
|  |  | RabSeq R | GATATYTGKCCYCATGGGAG |
|  | Actin | Lago Actin f | TTAAGCAATGTGTCGACGGGA |
|  |  | Lago Actin r | CCAGGGGTCAATGCCAATTTC |
|  | vWF | Lago vwr F | CTCGAAGTCCTGGATGAGGATG |
|  |  | Lago vwr R | CCATTTTGCTTTCTTCCCTGCT |
|  | Rabbit EVE | Lago N Flk vp1 f | TGGAGTTCGGTCTTGAACACT |
|  |  | lago n flk rep r | TCCAGGGTGTGCATTAGCAG |
|  |  | lago flk vp1 out f | AAAGGGAGAAAGCACCATAGGG |
|  |  | lago flk out rep r | CCTGTGACATCATTGTCAGGGAAC |
| Vesper Bats |  |  |  |
|  | Mitochondrial | Vesp cytb f | ACACGAAAAATCACCGTTGTATTTC |
|  |  | Vesp cytb r | TAGAATATCAGCTTTGGGTG |
|  | Actin | Vesp Actin f | TCTTTTTGTCCCATTCC |
|  |  | Vesp Actin f | TTGTGTAAATATAATCATTGTC |
|  | vWF | vWF-A | CTGTGATGGTGTCAACCTCACCTGTGAAGCCTG |
|  |  | vWF-B | TCGGGGGAGCGTCTCAAAGTCCTGGATGA |
|  | Vesper EVE | Chiro flk pri 8.5 f | ACCTTCTAACAGACACATATTCCCATTAG |
|  |  | Chiro nest flk vp r | CATAAATTGCAGTGGGATACTCCATTTGC |
|  |  | rep-chiro flk rep f | CCAAAATATTTGTATCCAGGAACAACAGGC |
|  |  | rep-chiro flk rep r | AGCCAGGGTCGGGACTTCTTAACGGTAGACAAG |
