# Supplementary material for: Evolution of dependoparvoviruses across geological timescales—implications for design of AAV-based gene therapy vectors
Source: Virus Evol. 2020 May 22;6(2):veaa043. doi: 10.1093/ve/veaa043 (PMC7474932; doi:10.1093/ve/veaa043)
Supplement: veaa043_Supplementary_Data [file ve_6_2_veaa043_s7.zip › S6 Table.docx]

S6 Table- Sequences Used for Circulating Dependoparvovirus Reference Genomes

| Circulating Dependoparvovirus Reference Genomes | |
| --- | --- |
| AAV1 | NC_002077.1 Adeno-associated virus - 1, complete genome |
| AAV2 | NC_001401.2 Adeno-associated virus - 2, complete genome |
| AAV3 | NC_001729.1 Adeno-associated virus - 3, complete genome |
| AAV3B | AF028705.1 Adeno-associated virus 3B, complete genome |
| AAV4 | DI393763.1 Adeno-associated virus - 4 |
| AAV5 | AF085716.1 Adeno-associated virus 5, complete genome |
| AAV6 | AF028704.1 Adeno-associated virus 6 |
| AAV7 | NC_006260.1 Adeno-associated virus - 7, complete genome |
| AAV8 | NC_006261.1 Adeno-associated virus - 8, complete genome |
| AAV9 | AX753250.1 Adeno-associated virus 9 |
| AAV10 | LY408697.1 Adeno-associated virus 10 |
| AAV11 | AY631966 Adeno-associated virus 11 |
| AAV12 | DQ813647 Adeno-associated virus 12 |
| Avian AAV DA-1 | AY629583.1 Avian adeno-associated virus strain DA-1 |
| Bat AAV YNM | NC_014468.1 Bat adeno-associated virus YNM |
| Bearded Dragon Parvovirus | NC_027429.1 Bearded dragon parvovirus strain |
| Bovine AAV | NC_005889.1 Bovine adeno-associated virus |
| California Sea Lion AAV1 | NC_038539.1California sea lion adeno-associated virus 1 |
| Caprine AAV | DQ335246 Caprine Adeno-associated virus -Go.1 |
| Murine AAV1 | MF416383.1 Murine adeno-associated virus 1 |
| Murine AAV2 | MF416384.1 Murine adeno-associated virus 2 |
| Muscovy Duck AAV | NC_040671 Adeno-associated virus isolate MHH-05-2015 |
| Muscovy Duck Parvovirus | NC_006147.2 Muscovy duck parvovirus |
| Simian AAV Cg34 | KT984498.1 Simian Adeno-associated virus isolate Cg34 |
| Snake Parvovirus | NC_006148.1 Snake parvovirus 1 |
