# Supplementary material for: Evolution of dependoparvoviruses across geological timescales—implications for design of AAV-based gene therapy vectors
Source: Virus Evol. 2020 May 22;6(2):veaa043. doi: 10.1093/ve/veaa043 (PMC7474932; doi:10.1093/ve/veaa043)
Supplement: veaa043_Supplementary_Data [file ve_6_2_veaa043_s7.zip › S7 Table.docx]

S7 Table- EVE-Positive Cell Lines

| EVE-Positive Cell Lines | |  | | |
| --- | --- | --- | --- | --- |
| Cell Line Name | Species | | Order | Source |
| TuTr | *Tursiops truncatus* | | Cetacea | Sand Diego Global, Lab # 12580 |
| Fin | *Balaenoptera physalus* | | Cetacea | Sand Diego Global, Lab # 17518 |
| RK13 | *Oryctolagus cuniculus* | | Lagomorpha | ATCC® CCL-37 |
| SF1 | *Sylvilagus floridanus* | | Lagomorpha | ATCC® CCL-68 |
| MVI-it | *Myotis velifer incautus* | | Chiroptera | Discontinued ATCC product CRL-6012 |
