# Supplementary material for: Evolution of dependoparvoviruses across geological timescales—implications for design of AAV-based gene therapy vectors
Source: Virus Evol. 2020 May 22;6(2):veaa043. doi: 10.1093/ve/veaa043 (PMC7474932; doi:10.1093/ve/veaa043)
Supplement: veaa043_Supplementary_Data [file ve_6_2_veaa043_s7.zip › S9a-c Fig.docx]

- S9a-c Fig- Vesper Bat Phylogenetic Trees generated using RAxML and 500 bootstrapping replicates for:
  - S9a Fig-Vesper Bat Eve sequences
  - S9b Fig-Vesper Bat Control Genes composed of a concatemer sequence containing mitochondrial Cytb, Actin intron, and vwf sequences.
  - S9c Fig-Vesper Bat Control+EVE composed of a concatemer sequence containing mitochondrial Cytb, Actin intron, vwf and EVE sequences.

S9a Fig

S9b Fig

S9c Fig
