# Supplementary figures and images for: Evolution of dependoparvoviruses across geological timescales—implications for design of AAV-based gene therapy vectors
Source: Virus Evol. 2020 May 22;6(2):veaa043. doi: 10.1093/ve/veaa043 (PMC7474932; doi:10.1093/ve/veaa043)

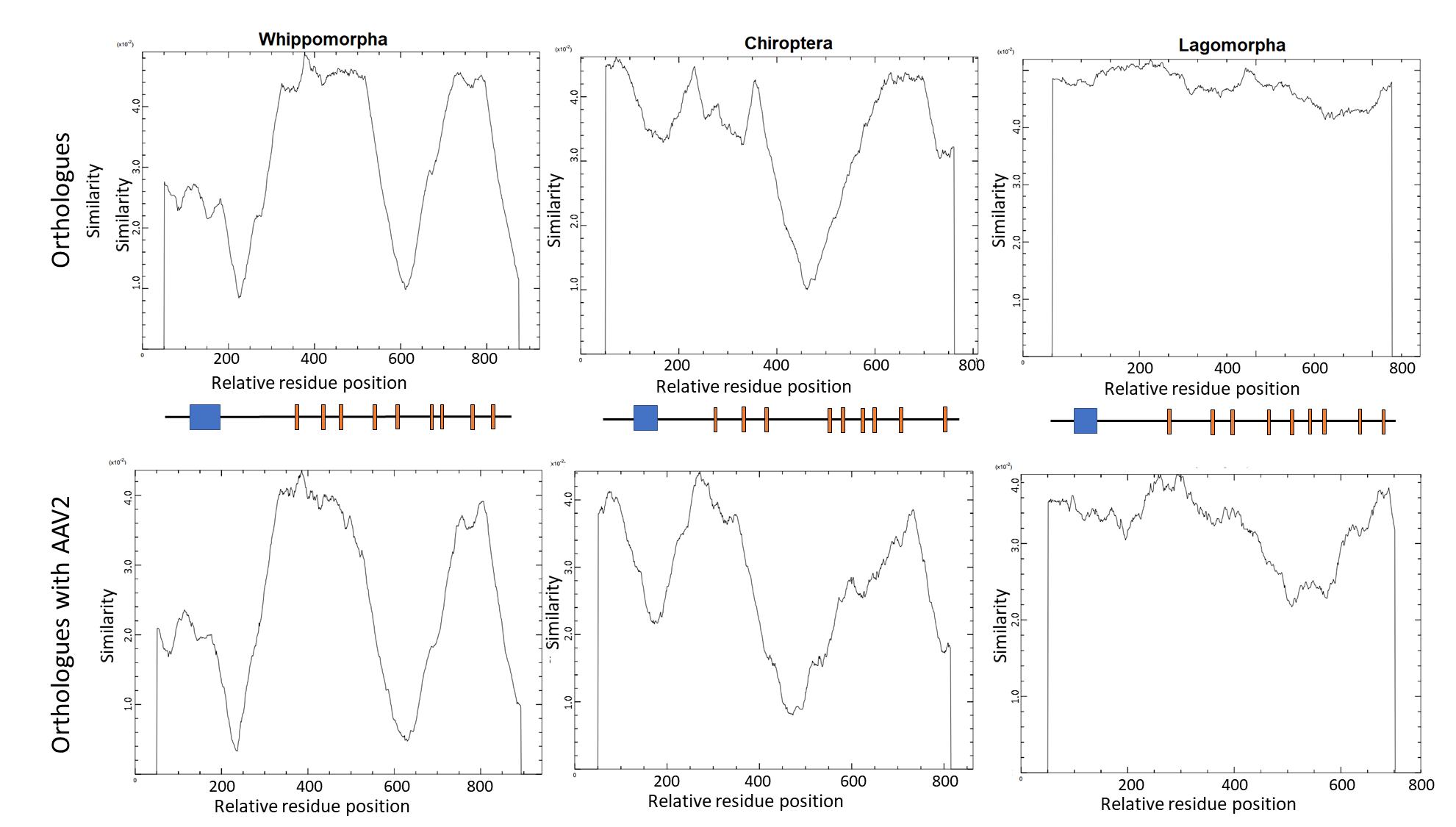

Supplement: veaa043_Supplementary_Data [file ve_6_2_veaa043_s7.zip › FigS6.tif]
